# Dysregulation of NK cell subsets and phenotypes in COVID-19 patients with comorbid type 2 diabetes

**DOI:** 10.1042/CS20243133

**Published:** 2025-06-23

**Authors:** Kang Lei, Wenqi Fan, Ting Zhong, Xinyu Li, Rong Tang, Bin Zhao, Xia Li

**Affiliations:** 1 Department of Endocrinology, The Second Affiliated Hospital, University of South China, Hengyang, Hunan , 421001, China; 2 National Clinical Research Center for Metabolic Diseases, Key Laboratory of Diabetes immunology, Ministry of Education, and Department of Metabolism and Endocrinology, The Second Xiangya Hospital of Central South University, Changsha, Hunan, China; 3 CSU-Sinocare Research Center for Nutrition and Metabolic Health, Xiangya School of Public Health, Central South University, Changsha, Hunan, China; 4 Furong Laboratory, Changsha, Hunan, China

**Keywords:** COVID-19, flow cytometry, immunology, immunometabolism, innate immunity, NK cells, type 2 diabetes

## Abstract

Recent studies have linked natural killer (NK) cells to COVID-19. However, the role of NK cells in COVID-19 patients complicated with type 2 diabetes (T2D) remains unexplored. Our findings indicate no significant differences in the frequency or immunophenotype of total NK cells and the CD56^bright^ CD16^-^ subset among COVID-19 patients, T2D patients, and healthy individuals. Patients with severe COVID-19 had a greater prevalence of CD56^dim^ CD16^-^ cells subset and a lower prevalence of CD56^dim^ CD16^+^ cells subset, with these trends being even more pronounced in those with comorbid T2D. The proportion of CD56dim CD16+ cell subset exhibited a significant negative correlation with both interleukin-6 levels and the duration of hospital stay. Furthermore, when COVID-19 patients were compared with patients with T2D or control subjects, a trend was noted toward increased expression of CD69, KIR, and CD52 and decreased expression of CD226, NKG2D, and CD62L. These immunophenotypic changes were particularly accentuated in COVID-19 patients with comorbid T2D. Importantly, the CD56^dim^ CD16^+^ cells subset emerges as a substantial predictor of COVID-19 severity. Together, COVID-19 patients exhibit alterations in NK cell subsets, with aggravated dysregulation in individuals with T2D, and the CD56^dim^ CD16^+^ cells subset may serve as an indicator of COVID-19 severity.

## Introduction

In 2021, 529 million people worldwide have diabetes, with type 2 diabetes (T2D) representing a staggering 96% of these cases [1]. T2D is marked by insulin resistance and beta-cell dysfunction [2]. Projected to surge by nearly 61.2% within the next three decades, T2D is expected to affect over 1.27 billion individuals. This alarming forecast underscores the substantial challenges associated with the escalating prevalence and mortality rates, as well as the potential for reduced life expectancy [1]. However, the underlying immunological mechanisms involved in T2D remain to be fully elucidated.

Viral infections, like cytomegalovirus and hepatitis C, among other environmental factors, are increasingly acknowledged for their role in the development of T2D [3,4]. The pathogen causing the COVID-19 pandemic is SARS-CoV-2, which affects not only the respiratory system but also the pancreas, affecting glucose metabolism [5]. Accumulating evidence indicated a substantial increase in the occurrence of T2D during the pandemic, with rates at least doubling compared with prepandemic levels [6–8]. COVID-19 has also been linked to disruptions in glycometabolic regulation, a condition that may persist for up to two months post-recovery, underscoring the lingering effects of the virus on metabolic health. Moreover, patients with T2D have been found to experience longer hospital stays and exhibit worse respiratory parameters, suggesting that SARS-CoV-2 may exacerbate the already heightened proinflammatory state characteristic of T2D [9]. The COVID-19 pandemic has served to further illuminate the intricate nexus between viral infections and the progression of T2D, highlighting the multifaceted nature of this disease.

As pivotal elements of the immune system’s defense against viral infections [10], natural killer (NK) cells represent 5–19% of lymphocytes in the peripheral blood. These cells are categorized into three unique subsets based on the cell surface density of CD56 and the expression of CD16: CD56^dim^ CD16^+^ cells subset, also known as effector NK (NKeff) cells, accounts for about 90% of the NK cells in circulation and is known for their strong cytotoxic capabilities; a small fraction, roughly 10%, are CD56^bright^ CD16^-^ cells subset and CD56^dim^ CD16^-^ cells subset. CD56^bright^ CD16^-^ cells subset is predominantly engaged in the production and release of cytokines, and CD56^dim^CD16^-^ cells subset is a less understood non-classical subset [11]. The function of NK cells in the context of COVID-19 is increasingly clarified. Research has demonstrated that NK cells in COVID-19 patients are significantly activated but also exhibit signs of exhaustion. They undergo significant phenotypic and functional alterations, which often result in diminished cytotoxic capabilities and impaired cytokine production [12–14]. A prior study indicated that T2D correlates with impaired NK cell function and increased apoptosis, possibly due to the up-regulation of Tim-3 [15]. Nevertheless, the influence of NK cells among patients with both COVID-19 and T2D—whether they are beneficial or pathological—has remained largely unclear.

To bridge this gap, this study focused on individuals with T2D complicated with COVID-19 to identify the expression and distribution of NK cell subsets. We assessed the prevalence of various NK cell subsets and explored the correlations between these subsets and the clinical features of patients with COVID-19 and T2D. We further examined the phenotypic changes in NK cell subsets and the indicators of COVID-19 disease severity. We discovered that COVID-19 patients display changes in NK cell subsets, with more severe dysregulation in those with T2D, suggesting that the CD56^dim^ CD16^+^ subset may be an indicator of COVID-19 severity. In summary, our study identified a significant deep shift in the immune profile of NK cells among COVID-19 patients with T2D, which may represent a critical factor influencing the clinical outcomes in these patients.

## Materials and methods

### Study subjects

We conducted this study at the Second Xiangya Hospital of Central South University (CSU), enrolling 91 patients hospitalized with COVID-19 between December 20, 2022, and January 20, 2023. To provide a comprehensive comparison, we also recruited 35 healthy individuals and 33 patients diagnosed with T2D who were not hospitalized with COVID-19. T2D was diagnosed in accordance with the World Health Organization’s 1999 criteria for diabetes [16], ensuring that patients tested negative for islet autoantibodies. For the healthy individuals, a necessary condition was the lack of a diabetic family history. Participants with pre-existing conditions known to significantly alter immune phenotypes were excluded, including cardiovascular disease, chronic obstructive pulmonary disease, autoimmune disorders under immunosuppressive therapy, and chronic kidney disease (eGFR<30 ml/min/1.73 m²) (Supplementary Figure S1). COVID-19 cases were verified by identifying SARS-CoV-2 via reverse transcription polymerase chain reaction from respiratory specimens. The severity of COVID-19 cases clinically was graded utilizing the established illness severity categories from the National Institutes of Health, correlating with a numerical scoring system ranging from 1 to 5 [17], as detailed below:

1: Asymptomatic.

2: Mild.

3: Moderate: characterized by pneumonia with oxygen saturation levels of 94% or higher.

4: Severe, identified by pneumonia with a respiratory rate greater than 30, oxygen saturation below 94%, a PaO2/FiO2 ratio less than 300, and lung infiltration exceeding 50%.

5: Critical, representing cases with respiratory failure, shock, or multiple organ dysfunction.

For the purposes of our analysis, patients were categorized as follows: scores of 1–3 were classified as ‘mild’, while scores of 4–5 were deemed ‘severe’.

### Clinical features and biochemical measurements

Body height, weight, and blood pressure were recorded by clinical physicians. BMI was subsequently calculated using the formula where weight divided by height squared. Additionally, a routine blood examination, assessments of liver and kidney function, blood lipid profiles, and inflammatory markers were conducted within the Department of Clinical Laboratory at the Second Xiangya Hospital of CSU.

### Isolation of peripheral blood mononuclear cells and flow cytometry

Following blood collection in sodium heparin tubes, the samples were promptly processed within a 3-hour window. Using the standard Ficoll-Paque Plus density-gradient centrifugation method, peripheral blood mononuclear cells (PBMCs) were isolated from the total blood volume and then cryopreserved at −196°C for subsequent analysis. Prior to flow cytometry analysis, PBMCs were transferred to cytometry tubes. In the process of identifying and phenotyping NK subsets, the following panel of antibodies was assembled: CD3, CD56, CD16, CD226, CD52, NKG2D, CD62L, CD69, and KIR2DL1/S1/S3/S5, along with Fixable Viability Stain 780, all in strict adherence to the protocols provided by Biolegend or BD Pharmingen. NK cells are categorized into three distinct subsets: CD56^bright^ CD16^-^ cells subset, CD56^dim^ CD16^-^ cells subset, and NKeff cells subset (CD56^dim^ CD16^+^ NK). Flow cytometry analysis was conducted utilizing an LSR II instrument by BD Biosciences, with data analysis handled by FlowJo version 10.8.1 software, which was developed by Treestar in San Carlos, CA, USA. For an overview of the gating strategy employed, refer to Supplementary Figure S2. A comprehensive list of the flow cytometry analysis panel is detailed in Supplementary Table S1.

### *In vitro* co-culture of NK cells and IL-6 and glucose

To determine the frequency of NK cell subsets and the intracellular expression of granzyme B (GZMB), perforin, Interferon-γ (IFN-γ), and IL-6, NK cells were isolated from PBMCs of healthy donors and seeded into 96-well plates containing 200 μl of complete RPMI 1640 medium. The cells were then stimulated with IL-6 (10 ng/ml; R&D) alone or in combination with tocilizumab (TCZ; anti-human IL-6R, 50 μg/ml; MCE) for 48 hours [18]. In parallel experiments, NK cells were incubated for 24 hours in media with varying glucose concentrations: 5 mmol/L (standard glucose concentration for general cell culture) and two diabetogenic glucose concentrations (15 mmol/L and 25 mmol/L) to simulate a high-glucose environment [19]. After stimulation, the cells were surface-stained with monoclonal antibodies (mAbs) targeting CD3 and CD56, fixed and permeabilized using Cytofix/Cytoperm™ buffers, and subsequently stained intracellularly with fluorochrome-conjugated antibodies specific for GZMB, perforin, IFN-γ, and IL-6. Flow cytometry was employed for data acquisition and analysis.

### Statistical analysis

Statistical analysis was performed using GraphPad Prism version 9.0 and R version 4.3.1 software. For continuous variables, data were displayed as the mean with standard deviation or as the median with interquartile ranges from the 25th to the 75th percentile. Categorical variables were expressed as frequencies and their corresponding percentages. Student’s *t*-test was applied to assess continuous variables across two groups, and one-way analysis of variance was used for assessing differences among three or more groups. Fisher’s exact test or a χ2 test was utilized for the analysis of categorical variables where appropriate. The Mann–Whitney *U* test was utilized for nonparametric assessments. Additionally, univariate COX regression analysis was employed for investigating the link between the severity of COVID-19 and clinical characteristics. The relationship between the frequencies of NK cell subsets and clinical features in COVID-19 patients was determined using Pearson’s correlation test. To address potential confounding effects in intergroup comparisons, adjustment for the differences in age and sex was carried out with logistic regression for categorical variables, analysis of covariance for parametric variables, and quantile regression for nonparametric variables. For multidimensional immunophenotypic and functional comparisons, adjustments were conducted by multiple comparisons. Statistical significance was ascertained with a *P* value cut-off at less than 0.05.

## Results

### Clinical features of study population throughout the study

Clinical features of healthy controls and COVID-19 patients are shown in Table 1. Among the 91 COVID-19 patients, 57 were diagnosed with mild forms of the disease, whereas 34 had severe cases. Patients exhibiting severe manifestations of COVID-19 were significantly older (70 years vs. 59 years) and experienced longer hospital stays (14 days vs. 10 days) compared with those with milder symptoms. Additionally, they presented a higher rate of ICU admissions (9% vs 0%), increased mortality rates (18% vs 0%), and exhibited more pronounced inflammatory responses. Notably, severe cases exhibited a significant impairment in liver and kidney function. Strikingly, the rate of comorbid diabetes was substantially higher in patients with severe COVID-19, with 71% of this group affected, compared with 44% in the mild COVID-19 group.

**Table 1:** Clinical characteristics of the participants included in the study.

Demographics and clinical characteristics	HC, *N* = 35	Mild, *N* = 57	Severe, *N* = 34	Age- and sex-adjusted *P* value^1^
Gender (male)	14 (40.0%)	26 (45.6%)	26 (76.5%) ^2^^,6^	
Age (years)	59 (55, 67)	59 (47, 74)	72 (67, 76) ^4^	
Height (cm)	161 ± 9	161 ± 9	163 ± 8	0.961
Weight (Kg)	60 (55, 66)	59 (51, 71)	65 (56, 70)	0.243
BMI (Kg/m^2^)	23.02 ± 2.54	23.31 ± 3.08	24.38 ± 3.38	0.723
Length of stay in hospital (days)	NA	10.0 (7.0, 13.0)	14.0 (10.3, 20.3)	0.002
Combined with diabetes (%)	NA	25 (44%）	24 (71%）^5^	< 0.001
ICU admission (%)	NA	0 (0%）	3 (9%）^5^	0.996
Mortality (%)	NA	0 (0%）	6 (18 %) ^5^	0.997
WBC (x 10^9^ /L)	6.4 (5.2, 7.1)	6.4 (4.9, 8.3)	7.2 (6.2, 10.2)	0.248
Neutrophils (x 10^9^ /L)	4.0 (3.4, 4.9)	4.3 (2.6, 6.5)	6.1 (4.0, 8.5) ^4^	0.133
Lymphocytes (x 10^9^ /L)	1.38 (1.11, 1.74)	1.36 (0.95, 1.86)	0.79 (0.46, 1.33)^4^^,6^	0.034 HC vs. mild: 0.942 HC vs. severe: 0.034 Mild vs. severe: 0.069
Monocytes (x 10^9^ /L)	0.36 (0.30, 0.44)	0.36 (0.30, 0.44)	0.36 (0.30, 0.44)	0.885
Lactic	NA	2.16 (1.81, 3.20)	2.40 (2.21, 2.72)	0.697
ESR	NA	42 (34, 62)	49 (42, 66)	0.361
CRP (mg/L)	NA	25 (6, 46)	63 (14, 122) ^6^	< 0.001
PCT	NA	0.09 (0.03, 0.20)	0.13 (0.07, 0.60) ^6^	0.679
IL-6 (ng/ml)	NA	9 (4, 13)	44 (8, 89) ^7^	< 0.001
LDH (U/L)	NA	259 (202, 259)	325 (254, 371) ^7^	0.001
ALT (U/L)	17 (10, 29)	25 (17, 39)^2^	29 (17, 43) ^3^	0.058
AST (U/L)	19 (16, 22)	23 (16, 34)^2^	27 (17, 40) ^3^	0.093
TBIL (μmol/L)	9.6 (6.7, 11.3)	9.8 (7.5, 12.7)	8.8 (6.3, 10.3)	0.272
DBIL (μmol/L)	3.10 (2.20, 3.90)	3.70 (2.60, 4.60)	4.10 (2.90, 5.20) ^3^	0.085
BUN (μmol/L)	5 (4, 7)	5 (4, 6)	9 (5, 14) ^2,7^	0.148
Cr (μmol/L)	66 (53, 78)	69 (58, 82)	85 (65, 121) ^6,4^	0.074
UA (μmol/L)	214 (160, 281)	308 (234, 350)^4^	319 (249, 374) ^4^	< 0.001 HC vs. mild: 0.001 HC vs. severe: < 0.001 Mild vs. severe: 0.460
TG (mmol/L)	1.33 (1.12, 1.76)	1.39 (1.02, 2.51)	1.42 (1.03, 1.80)	0.691
CHOL (mmol/L)	4.19 ± 1.41	3.92 ± 1.19	3.73 ± 1.05	0.382
LDL-C (mmol/L)	2.64 ± 1.09	2.63 ± 0.86	2.26 ± 0.95	0.944
HDL-C (mmol/L)	0.89 (0.68, 1.25)	0.97 (0.75, 1.15)	0.92 (0.76, 1.05)	0.981
HbA1c (%)	5.61 (5.33, 5.93)	6.15 (5.57, 8.51) ^4^	7.34 (6.15, 8.11) ^4^	< 0.001 HC vs. mild: 0.140 HC vs. severe: < 0.001 Mild vs. severe: 0.073
25-(OH) VitD (nmmol/L)	41 (33, 59)	41 (30, 53)	43 (31, 53)	0.789
Antiviral treatment	NA	9 (15.8%)	18 (52.9%)^7^	0.002
Glucocorticoid	NA	10 (17.5%)	19 (55.9%) ^7^	0.024

Data are expressed as (n) for qualitative data, the mean ± standard deviation for parametric data and the median (interquartile ranges) for nonparametric data.

1 The first *P* values refer to comparisons across all groups, while the ones below refer to each separate two‐group comparison.

2 *P*<0.05 compared with HC.

3 *P*<0.01 compared with HC.

4 *P*<0.001 compared with HC.

5 *P*<0.05 compared with mild COVID-19.

6 *P*<0.01 compared with mild COVID-19.

7 *P*<0.001 compared with mild COVID-19.

ALT, alanine aminotransferase; AST, aspartate aminotransferase; BMI, body mass inde; BUN, blood urea nitrogen; CHOL, cholesterol; Cr, creatinine; CRP , C-reactive protein; DBIL, direct bilirubin; ESR, erythrocyte sedimentation rate; HDL, high-density lipoprotein; LDL, low-density lipoprotein; IL-6, interleukin-6; LDH, lactate dehydrogenase; NA, not applicable; PCT, procalcitonin; ICU, intensive care unit; TBIL, total bilirubin; TGs, triglycerides; UA, uric acid; WBC, white blood cell counts.

To ascertain the influence of T2D in the context of COVID-19, we expanded our study cohort to include an additional 33 patients diagnosed with T2D but free from COVID-19. Table 2 displays the clinical characteristics of the participants included in the study. Among the 91 COVID-19 patients, 49 had a prior diagnosis of T2D. Age and BMI were similar across healthy controls, individuals with only T2D, COVID-19 patients without T2D, and those with both COVID-19 and T2D. However, a comparison between COVID-19 patients with and without T2D revealed notable disparities in hospital stay duration (13 days vs. 9.0 days), mortality rates (13% vs. 0%), inflammatory levels (C-reactive protein: 46 ng/ml vs. 8 ng/ml), and HbA1c (8.05 vs 5.70 %). Notably, patients with T2D demonstrated higher levels of HbA1c compared with their non-diabetic counterparts. Furthermore, irrespective of T2D status, individuals with COVID-19 experienced a notably greater deterioration in liver and kidney function compared with those with T2D alone.

**Table 2:** Clinical characteristics of control subjects and patients with T2D and COVID-19.

Demographics and clinical characteristics	HC (*n* = 35)	T2D (*n* = 33)	COVID-19 without T2D (*n* = 42)	COVID-19 with T2D (*n* = 49)	Age- and sex-adjusted *P* value^1^
Gender (male)	14 (40.0%)	21 (63.6%)^2^	22 (52.4%)	30 (61.2%)^2^	
Age (years)	60 ± 10	59 ± 12	62 ± 19	64 ± 14	
Height (cm)	161 (154, 166)	167 (158, 169)	160 (156, 168)	162 (154, 168)	0.627
Weight (Kg)	60 (55, 66)	65 (59, 70)	59 (52, 70)	62 (53, 70)	0.426
BMI (Kg/m^2^)	23.0 ± 2.5	24.1 ± 3.2	24.3 ± 3.7	23.1 ± 2.8	0.168
Length of stay in hospital (days)	NA	NA	9.0 (7.0, 12.8)	13.0 (10.0, 17.0) ^10^	0.014
Disease severity index^2^					
2	NA	NA	19 (45.2%)	10 (20.4%)^8^	0.021
3	NA	NA	6 (14.3%)	11 (22.4%)	0.233
4	NA	NA	10 (23.8%)	18 (36.7%)	0.179
5	NA	NA	0 (%)	3 (6%)	0.997
ICU admission (%)	NA	NA	1 (2%)	2 (4%)	0.735
Mortality (%)	NA	NA	0 (0%)	6 (13 %)^8^	0.997
WBC ( × 10^9^ /L)	6.36 (5.22, 7.09)	6.35 (5.38, 7.74)	7.18 (5.55, 9.16)	6.57 (5.15, 8.97)	0.721
Neutrophils ( × 10^9^ /L)	4.04 (3.40, 4.90)	3.98 (3.43, 5.40)	4.76 (2.57, 7.28)	4.64 (3.61, 6.92)	0.252
Lymphocytes ( × 10^9^ /L)	1.38 (1.11, 1.74)	1.65 (1.27, 2.01)	1.35 (0.90, 1.79) ^5^	0.90 (0.53, 1.65) ^3,7^	0.117
Monocytes ( × 10^9^ /L)	0.36 (0.30, 0.44)	0.44 (0.34, 0.54)	0.42 (0.30, 0.58)	0.37 (0.27, 0.51)	0.947
Lactic	NA	NA	2.12 (1.82, 2.45)	2.48 (2.10, 2.96) ^8^	0.031
ESR	NA	NA	42 (27, 56)	50 (40, 62) ^8^	0.151
CRP (mg/L)	NA	NA	8 (4, 27)	46 (22, 109) ^10^	< 0.001
PCT	NA	NA	0.06 (0.04, 0.15)	0.12 (0.05, 0.39)	0.618
IL-6 (ng/ml)	NA	NA	5 (4, 10)	17 (9, 77) ^10^	0.116
LDH (U/L)	NA	NA	228 (186, 293)	305 (230, 378) ^8^	0.037
ALT (U/L)	17 (10, 29)	19 (14, 28)	25 (17, 47)^2^	28 (17, 40)^2^	0.005 I vs. II:0.982 I vs. III:0.183 I vs. IV:0.096 II vs. III:0.130 II vs. IV:0.050 III vs. IV: 0.893
AST (U/L)	19 (16, 22)	17 (14, 26)	25 (16, 35) ^2,5^	25 (17, 40)^3^^,6^	0.058
TBIL (umol/L)	9.6 (6.7, 11.3)	7.9 (6.6, 10.1)	9.7 (7.5, 12.5)	9.0 (6.2, 12.0)	0.861
DBIL (umol/L)	3.10 (2.20, 3.90)	3.10 (2.30, 4.00)	3.50 (2.60, 4.80)^2^	4.00 (2.80, 5.10) ^3,5^	0.056
BUN (mmol/L)	5.3 (4.3, 7.5)	5.8 (4.2, 6.7)	5.1 (3.6, 6.3)	6.8 (4.8, 12.4) ,^2^^,8^	0.376
Cr (umol/L)	66 (53, 78)	73 (58, 85)	70 (59, 79)	82 (59, 122) ^3^^,^^8^	0.061
UA (umol/L)	214 (160, 281)	272 (194, 349)	305 (249,356) ^3^	321 (232, 373) ^4^	< 0.001 I vs. II:0.054 I vs. III:0.015 I vs. IV: < 0.001 II vs. III:0.390 II vs. IV:0.065 III vs. IV: 0.396
TG (mmol/L)	1.33 (1.12, 1.76)	2.17 (1.57, 3.76) ^4^	1.26 (0.93, 2.27) ^6^	1.41 (1.13, 1.94) ^6^	0.723
CHOL (mmol/L)	4.19 ± 1.41	4.60 ± 1.14	3.99 ± 1.27^5^	3.75 ± 1.02^6^	0.208
LDL-C (mmol/L)	2.66 (1.67, 3.21)	3.00 (2.22, 3.46)	2.56 (2.15, 3.19)	2.27 (1.63, 2.92) ^5,8^	0.078
HDL-C (mmol/L)	0.89 (0.68, 1.25)	1.03 (0.84, 1.27)	1.00 (0.75, 1.31)	0.90 (0.74, 1.05)	0.908
HbA1c (%)	5.61 (5.33, 5.93)	8.49 (7.50, 10.05) ^4^	5.70 (5.32, 6.04) ^7^	8.05 (7.19, 9.92) ^4,10^	0.002 I vs. II: < 0.001 I vs. III:0.778 I vs. IV: < 0.001 II vs. III: < 0.001 II vs. IV:0.849 III vs. IV: < 0.001
25-(OH)VitD (nmol/L)	41 (33, 59)	38 (30, 49)	44 (38, 53)	40 (29, 53)	0.952
Antiviral treatment	NA	NA	13 (31.0%)	14 (28.6%)	0.789
Glucocorticoid	NA	NA	18 (42.9%)	11 (22.4%)^8^	0.016

Data are expressed as (n) for qualitative data, the mean ± standard deviation for parametric data and the median (interquartile ranges) for nonparametric data.

1 The first *P* values refer to comparisons across all groups, while the ones below refer to each separate two‐group comparison.

2 *P*<0.05 compared with HC.

3 *P*<0.01 compared with HC.

4 *P*<0.001 compared with HC.

5 *P*<0.05 compared with T2D.

6 *P*<0.01 compared with T2D.

7 *P*<0.001 compared with T2D.

8 *P*<0.05 compared with HC.

9 *P*<0.01 compared with COVID-19 without T2D.

10 *P*<0.001 compared with COVID-19 without T2D.

ALT, alanine aminotransferase; AST, aspartate aminotransferase; BMI, body mass inde; BUN, blood urea nitrogen; CHOL, cholesterol; Cr, creatinine; CRP, C-reactive protein; DBIL, direct bilirubin; ESR, erythrocyte sedimentation rate; HDL, high-density lipoprotein; LDL, low-density lipoprotein; IL-6, interleukin-6; LDH, lactate dehydrogenase; NA, not applicable; PCT, procalcitonin; ICU, intensive care unit; TBIL, total bilirubin; TGs, triglycerides; UA, uric acid; WBC, white blood cell counts.

### Altered peripheral CD56^dim^ CD16^-^ cells subset and NKeff cells subset frequencies in patients with COVID-19

**Figure 1:**
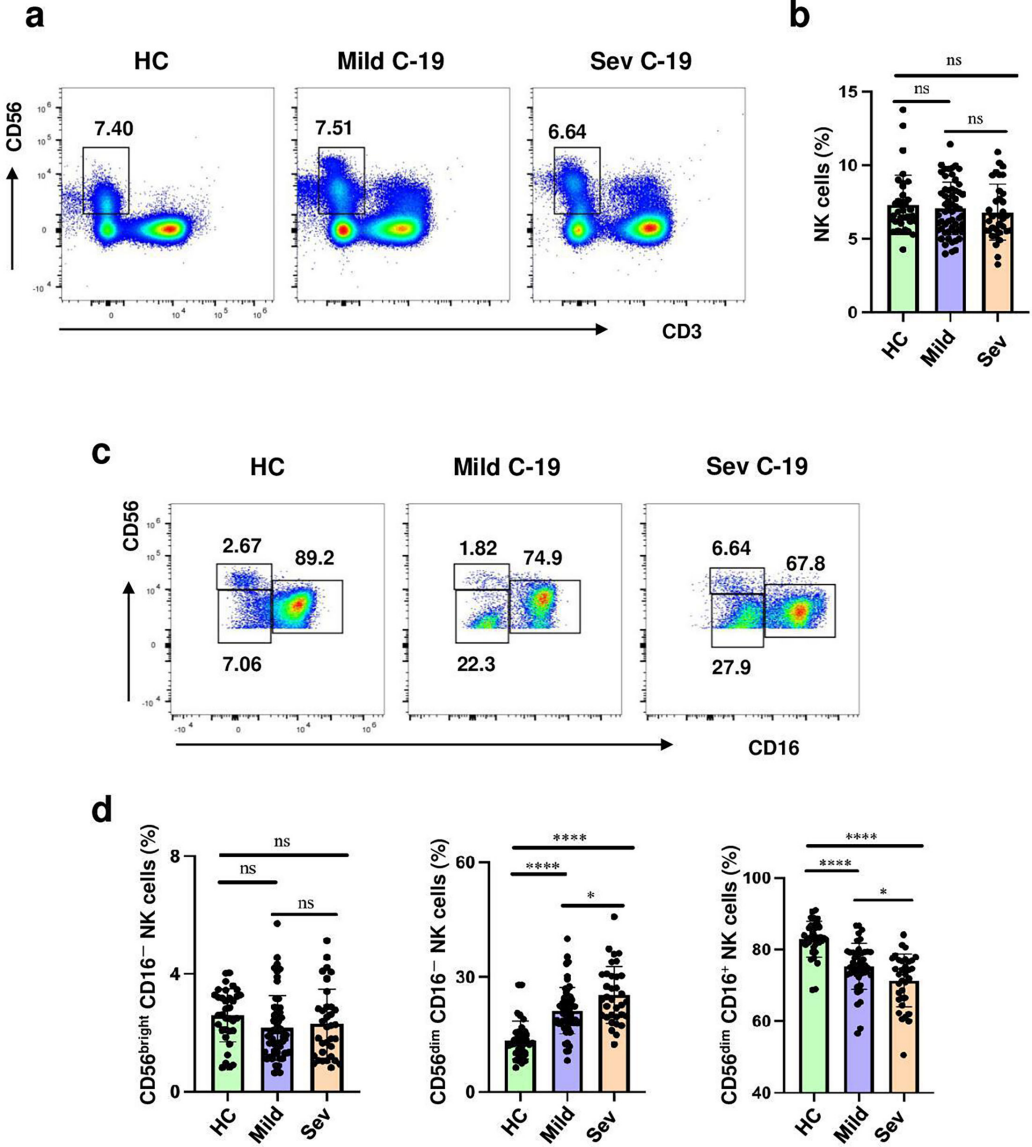
Altered peripheral CD56^dim^ CD16^-^ cells subset and NKeff cells subset frequencies in individuals with COVID-19. (**a**)The representative image showed the gating strategy for total NK cells and NK cell subsets. (**b**) Statistical analysis of total NK cells from healthy controls (HC) (*n* = 35), mild COVID-19 patients (*n* = 57), and severe COVID-19 patients (*n* = 34). (**c**) The NK cells subsets (CD56^bright^ CD16^-^ cells subset, CD56^dim^ CD16^-^ cells subset, NKeff cells subset) in HC, mild COVID-19 patients, and severe COVID-19 patients by flow cytometry analysis. (**e**) Statistical analysis of NK cells subsets from HC, mild COVID-19 patients, and severe COVID-19 patients. Horizontal bars represent the mean ± SD. ns, not significant, *P<0.05, **P<0.01, ***P<0.001, ****P<0.0001 by one-way ANOVA followed by adjustments for multiple comparisons.

In order to identify the prevalence of the three NK cell subsets among COVID-19 patients, detection of NK cells was conducted based on scatter-gated PBMCs, defined by CD3 and CD56, as well as CD16 and CD56. Figure 1 illustrates the representative plots for CD16 and CD56 expression on total NK cells across the various groups.

Regarding the overall NK cell count, significant differences were not observed in percentage referring to total PBMCs when comparing control subjects to patients with either mild or severe COVID-19 (all *P*>0.05). The NK cells were further categorized into three distinct subsets: CD56^bright^ CD16^-^ cells subset, CD56^dim^ CD16^-^ cells subset, and NKeff cells subset. The frequency of the CD56^bright^ CD16^-^ cell subset was similar among the three groups. However, a notable distinction was noted in the frequencies of the CD56^dim^ CD16^-^ cell subset and the NKeff cells subset. Compared with individuals with mild COVID-19 and control subjects, individuals suffering from severe COVID-19 exhibited a more elevated frequency of CD56^dim^ CD16^-^ cells and a reduced frequency of NKeff cells (all *P*<0.05). The prevalence of the NKeff cells subset progressively diminished from control subjects to patients with mild COVID-19 and then to those with severe COVID-19. In contrast, the prevalence of the CD56^dim^ CD16^-^ cell subset followed an opposite trend.

To account for potential confounding effects of disease progression, we stratified COVID-19 patients with T2D into short-term (≤5 years, *n* = 26) and long-term (>5 years, *n* = 23) cohorts based on T2D duration. Comparative analysis revealed no statistically significant differences (*P*>0.05 for all comparisons) in the frequencies of total NK cells or functionally defined subsets (CD56^bright^CD16^−^, CD56^dim^CD16^−^, and NKeff) between the two groups (Supplementary Figure S3).

In addition, to dissect medication-related influences on NK cell homeostasis (Supplementary Table S3), we performed a stratified analysis comparing patients receiving glucocorticoid therapy and/or antiviral agents with untreated controls. This systematic assessment (Supplementary Figure S4) demonstrated no statistically significant alterations in total NK cell frequencies or functionally distinct subpopulations (CD56^br^i^ghv^CD^−^, CD56^d^i^m^CD16^−^, and NKeff) between treatment-exposed and treatment-naïve groups (*P*>0.05 for all comparisons), suggesting these observed immunological profiles are independent of glucocorticoid or antiviral therapies.

### Frequency of CD56^dim^ CD16^-^ cells subset and NKeff cells subset correlate with clinical indicators of COVID-19 progression

**Figure 2:**
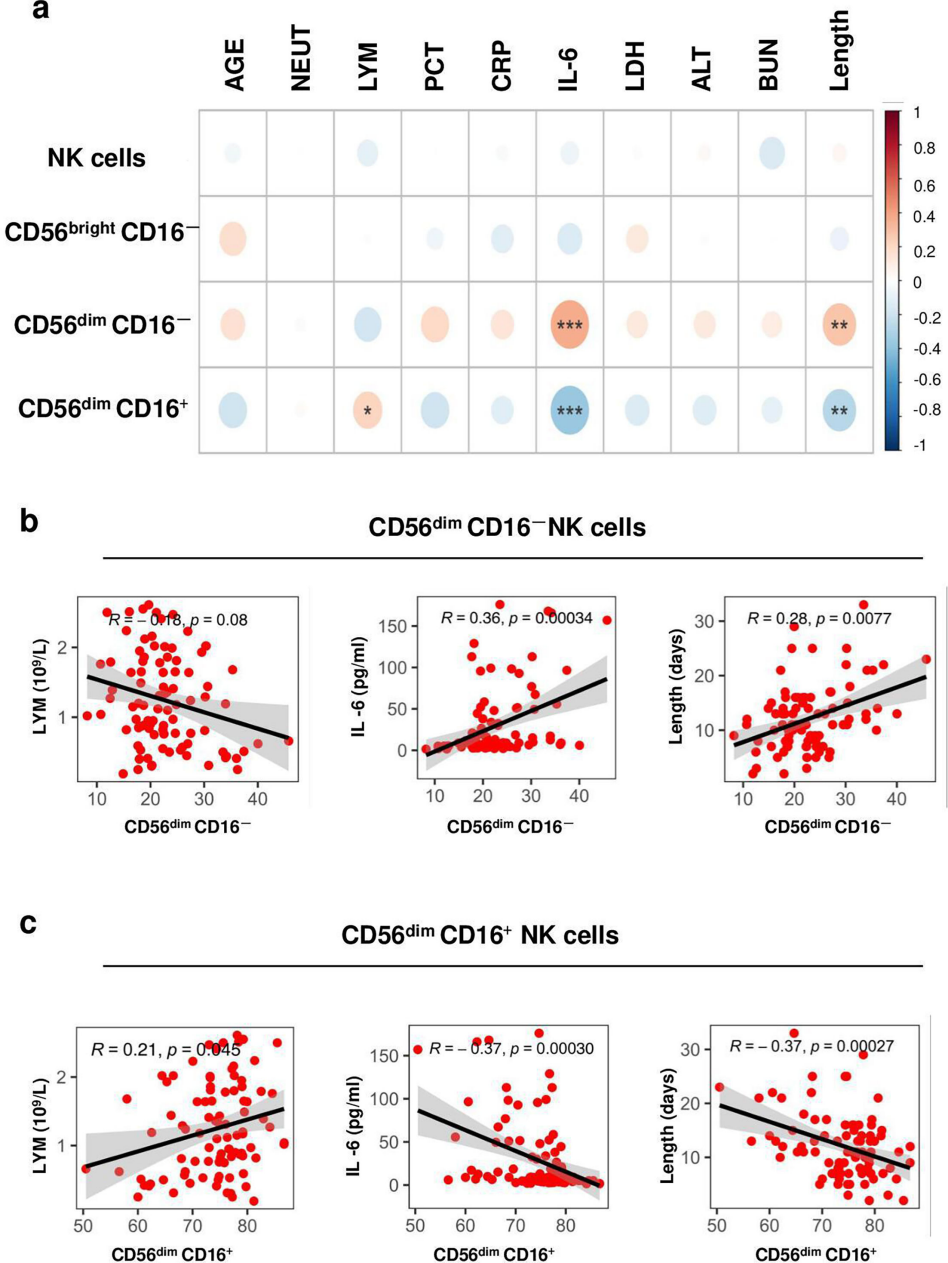
Frequencies of CD56^dim^ CD16^-^ cells subset and NKeff cells subset correlate with clinical indicators of COVID-19 progression. (**a**) Pearson’s correlation analysis of NK cells immunological parameters and clinical indicators in COVID-19 patients (*n* = 91). (**b, c**) Correlations between the frequency of CD56^dim^ CD16^-^ cells subset (**b**) and NKeff cells subset (**c**) and LYM, IL6, and the length of stay. Correlation was done by Pearson correlation coefficient and linear regression is shown with a 95% CI (gray area). ALT, alanine aminotransferase; BUN, blood urea nitrogen; CRP, C-reactive protein; IL-6, interleukin-6; LDH, lactate dehydrogenase; Length, length of stay in hospital; LYM, lymphocytes; NEUT, neutrophils; PCT, procalcitonin. *P<0.05, **P<0.01, ***P<0.001.

Referring to total NK cells and CD56^bright^ CD16^-^ cells subset, no significant correlations were identified in relation to clinical features, such as lymphocyte count, level of interleukin-6 (IL-6), and the duration of hospital stay. However, the percentage of NKeff cells subset demonstrated a significant negative correlation with both IL-6 levels (*R* = −0.37, *P*=0.0003) and the length of hospital stay (*R* = −0.37, *P*=0.00027). Additionally, a positive association was identified between the proportion of the NKeff cell subset and the lymphocyte count (*R* = 0.21, *P*=0.045). In contrast, the CD56^dim^ CD16^-^ cells subset exhibited an inverse relationship with these clinical features (Figure 2) (Supplementary Table S2). Changes in the frequency of CD56^dim^ CD16^-^ cells subset and NKeff cells subset are further amplified in COVID-19 patients comorbid T2D.

To elucidate the role of NK cells in patients with COVID-19 complicated by T2D, we stratified patients into four groups: those with T2D only, patients with COVID-19 only, and a group with both COVID-19 and T2D. The proportions of total NK cells and CD56^bright^ CD16^-^ cells subset were observed to be consistent across all four groups, without any significant differences between groups. COVID-19 patients with T2D had higher frequency of CD56^dim^ CD16^-^ cells subset and lower frequency of NKeff cells subset compared with patients with COVID-19 or T2D only (all *P*<0.05). The proportion of CD56^dim^ CD16^-^ cells and NKeff cells were comparable between control subjects and those with T2D (all *P*>0.05).

**Figure 3:**
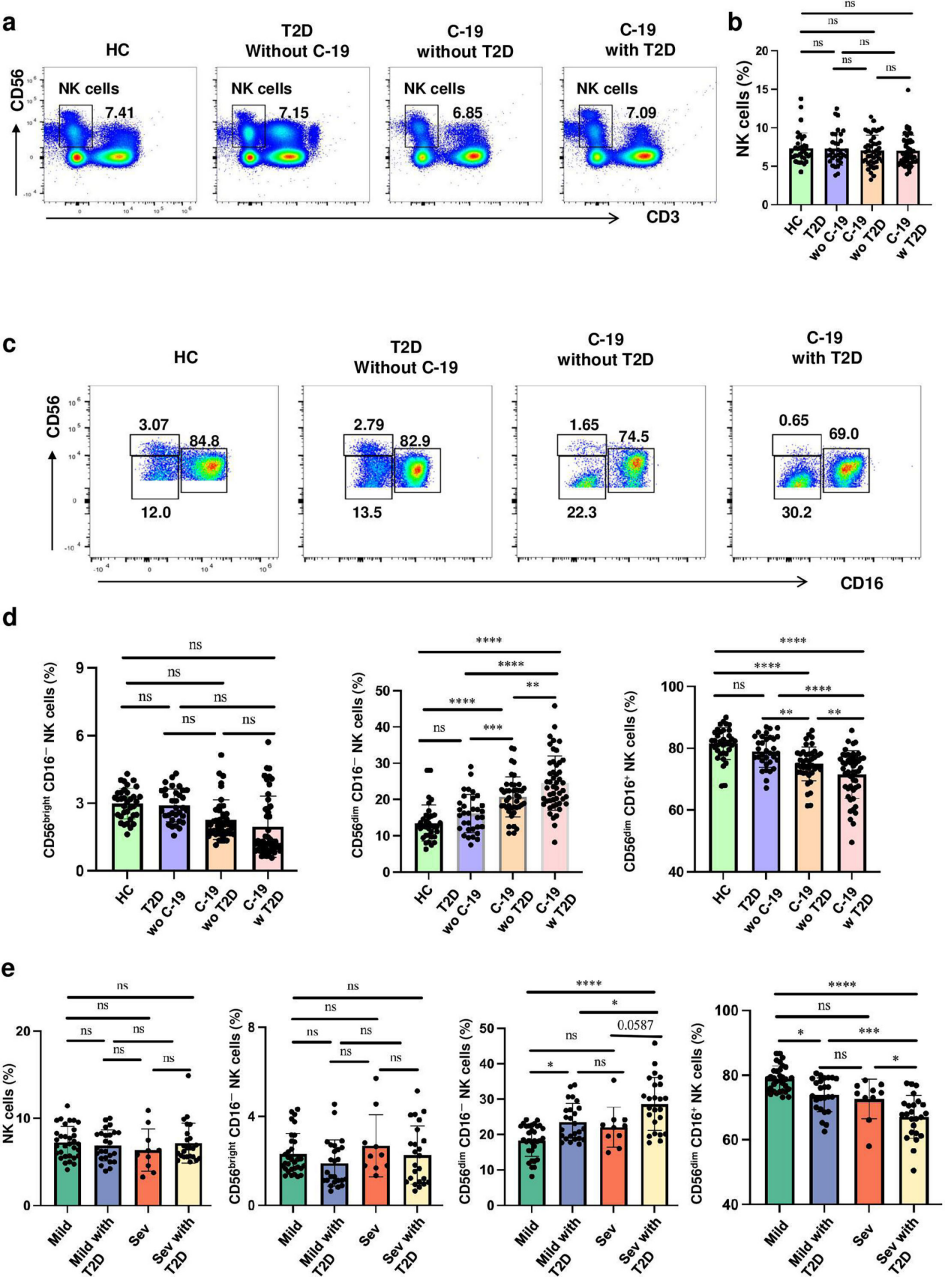
Changes in the frequencies of CD56^dim^ CD16^-^ cells subset and NKeff cells subset are further amplified in COVID-19 patients comorbid T2D. (**a**) Representative dot plots showing the frequency of total NK cells in healthy controls (HC), type 2 diabetes (T2D) without COVID-19, COVID-19 without T2D, and COVID-19 with T2D by flow cytometric analysis. (**b**) Statistical analysis of total NK cells from HC (*n* = 35), T2D without COVID-19 (n=33), COVID-19 without T2D (n=42), and COVID-19 with T2D (n=49). (**c**) Representative dot plots showing the frequency of CD56^bright^ CD16^-^ cells subset, CD56^dim^ CD16^-^ cells subset, NKeff cells subset in HC, T2D without COVID-19, COVID-19 without T2D, and COVID-19 with T2D by flow cytometric analysis. (**d**) Statistical analysis of NK cells subsets from HC, T2D without COVID-19, COVID-19 without T2D, and COVID-19 with T2D. (**e**) Statistical analysis of NK cells subsets from mild COVID-19 without T2D (*n* = 32), mild COVID-19 with T2D (n=25), severe COVID-19 without T2D (n=10), and severe COVID-19 withT2D (n=24). Horizontal bars represent the mean ± SD. ns, not significant, *P<0.05, **P<0.01, ***P<0.001, ****P<0.0001 by one-way ANOVA followed by adjustments for multiple comparisons.

Further, according to disease severity and T2D status, we categorized these patients into mild COVID-19, severe COVID-19, mild COVID-19 with T2D, and severe COVID-19 with T2D groups. Once more, no notable differences were identified in terms of the frequencies of NK cells and the CD56^bright^ CD16^-^ cells subset across the groups. However, for the NKeff cells subset, a reduced percentage was found in individuals experiencing severe COVID-19 and T2D when compared with other groups (all *P*<0.05). Additionally, patients experiencing mild COVID-19 and T2D exhibited a lower percentage compared with those with only mild COVID-19 (*P*<0.05). Conversely, the CD56^dim^ CD16^-^ cells subset displayed an opposite trend relative to the NKeff cells subset (Figure 3).

### Immunophenotype of NKeff cells subset undergoes strong alteration in COVID-19 patients comorbid T2D

**Figure 4:**
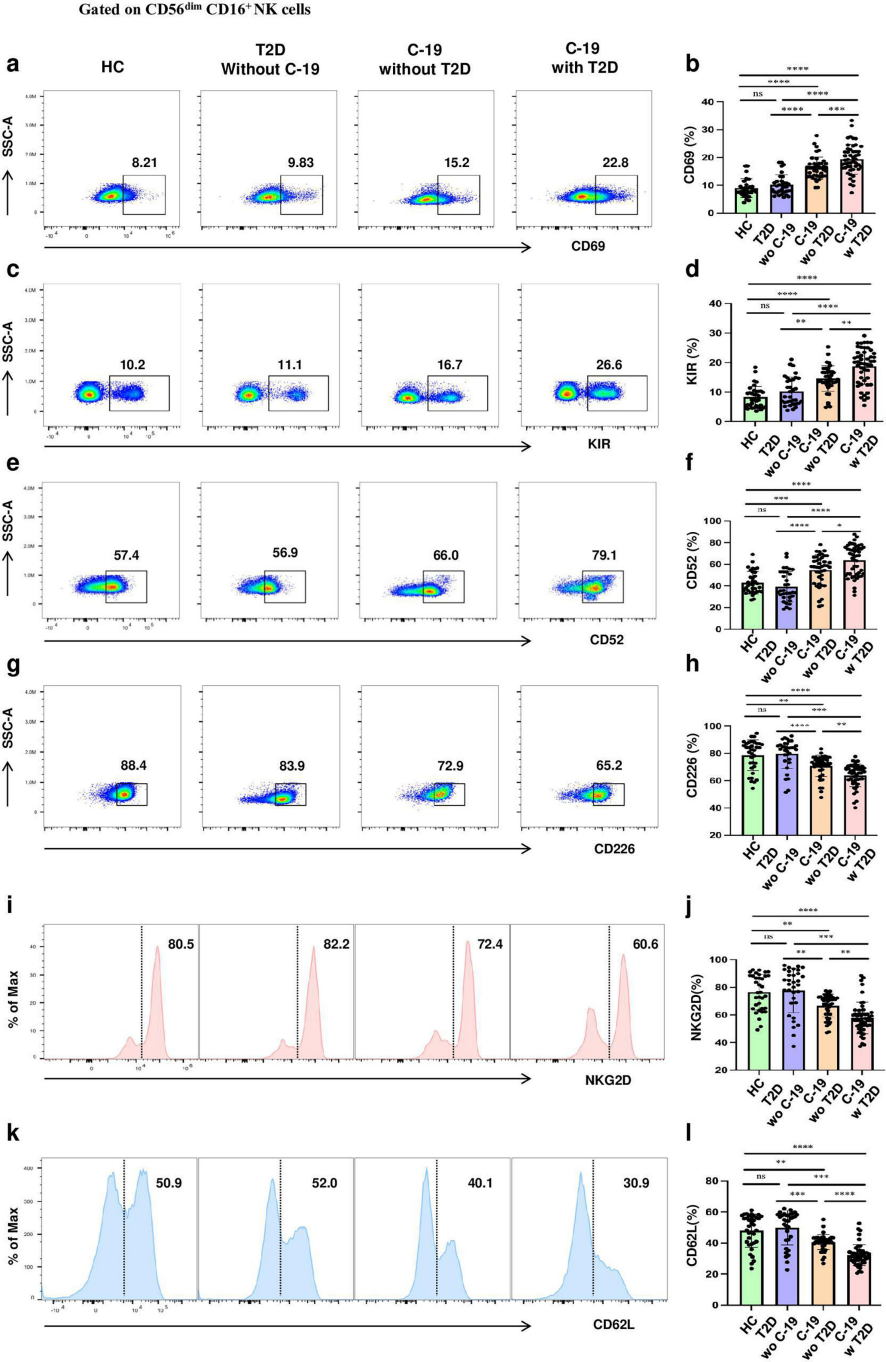
NKeff cells subset immunophenotype undergoes alterations in COVID-19 patients comorbid T2D. (**a, c, e, g, i, k**) Representative dot plots showing the expression of CD69 (**a**), KIR (**c**), CD52 (**e**), CD226 (**g**), NKG2D (**i**), and CD62L (**k**) by NKeff cells subset in HC, T2D without COVID-19, COVID-19 without T2D, and COVID-19 with T2D by flow cytometric analysis. (**b, d, f, h, j, l**). Statistical analysis of the expression of CD69 (**b**), KIR (**d**), CD52 (**f**), CD226 (**h**), NKG2D (**j**), and CD62L (**l**) by NKeff cells subset in HC (n=35), T2D without COVID-19 (n=33), COVID-19 without T2D (n=42), and COVID-19 with T2D (n=49). Horizontal bars represent the mean ± SD. ns, not significant, *P<0.05, **P<0.01, ***P<0.001, ****P<0.0001 by one-way ANOVA followed by adjustments for multiple comparisons.

**Figure 5:**
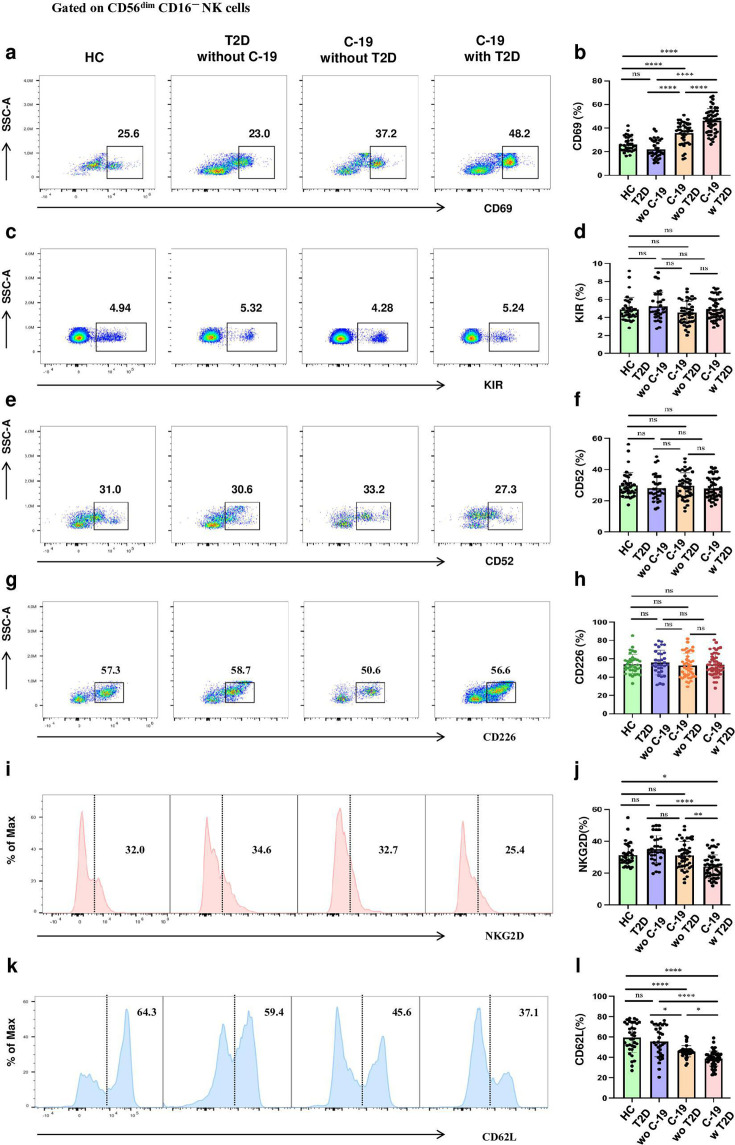
CD56^dim^ CD16^-^ cells subset immunophenotypes are strongly altered in patients with COVID-19 comorbid diabetes mellitus. (**a, c, e, g, i, k**) Representative dot plots showing the expression of CD69 (**a**), KIR (**c**), CD52 (**e**), CD226 (**g**), NKG2D (**i**), and CD62L (**k**) by CD56^dim^ CD16^-^ cells subsets in HC, T2D, COVID-19 without T2D, and COVID-19 with T2D by flow cytometric analysis. (**b, d, f, h, j, l**). Statistical analysis of the expression of CD69 (**B**), KIR (**d**), CD52 (**f**), CD226 (**h**), NKG2D (**j**), and CD62L (**l**) by CD56^dim^ CD16^-^ cells subsets in HC (*n* = 35), T2D (*n* = 33), COVID-19 without T2D (*n* = 42), and COVID-19 with T2D (*n* = 49). Horizontal bars represent the mean ± SD. ns, not significant, *P<0.05, **P<0.01, ***P<0.001, ****P<0.0001 by one-way ANOVA followed by adjustments for multiple comparisons.

Considering the significant alterations of NKeff cells subset in COVID-19 patients, a detailed assessment of the immunophenotype of NKeff cells subset was conducted, focusing on markers including CD69, KIR, CD52, CD226, NKG2D, and CD62L using flow cytometry. Our analysis revealed a trend towards elevated expression of CD69, KIR, and CD52, alongside diminished expression of CD226, NKG2D, and CD62L in patients who contracted COVID-19, when contrasted with those with T2D or control subjects. Importantly, these immunophenotypic changes were particularly pronounced in individuals who contracted COVID-19 and T2D (22.8%, 26,6%, 79.1%, 65.2%, 60.6% and 30.9%, respectively), as illustrated in Figure 4. Additionally, no significant distinctions were detected in the immunophenotypic profile of the CD56^bright^ CD16^-^ cells subset among the groups, as detailed in Supplementary Figure S5. Regarding to CD56^dim^ CD16^-^ cells subset, these cells of COVID-19 patients with T2D exhibited a higher CD69 expression and a lower NKG2D and CD62L expression in comparison with other groups (Figure 5).

### NKeff cells subset correlated with disease severity in COVID-19 patients

**Figure 6:**
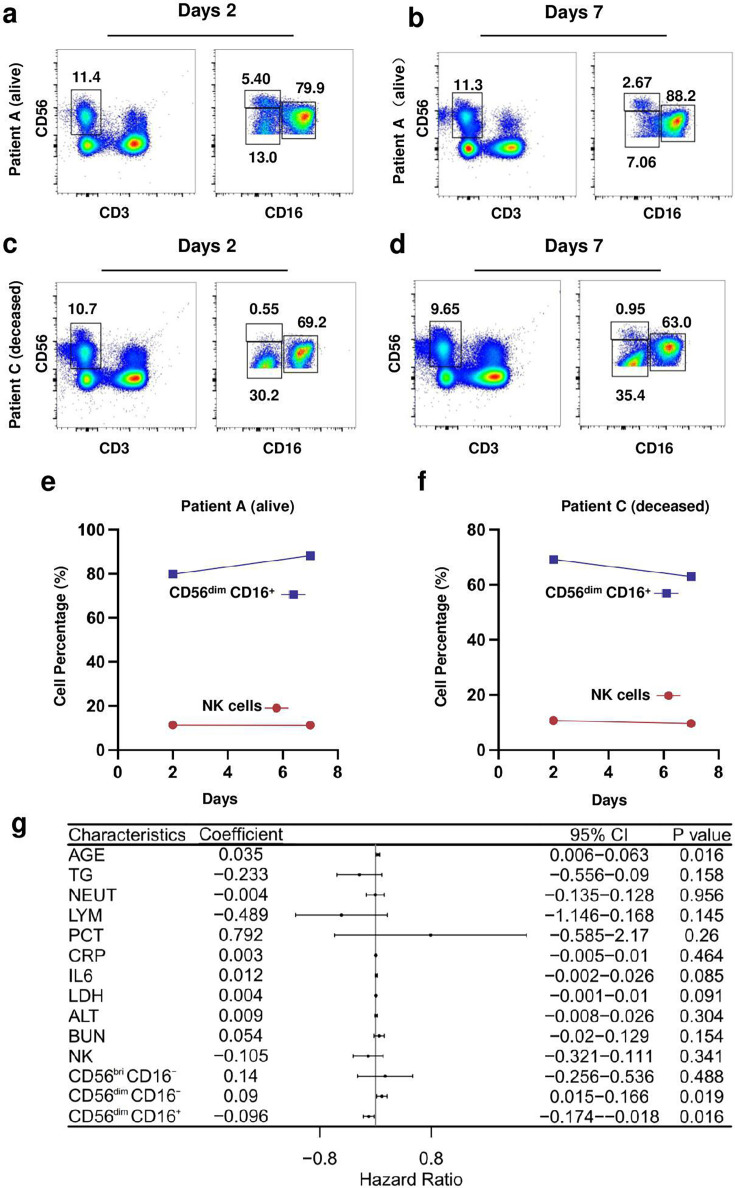
NKeff cells subset correlated with disease severity in COVID-19 patients. (**a, b, c, d, e, f**) the alternation trend of NKeff cells subset in the deceased patient (Patient C) and one recovery Severe COVID-19 patient (Patient A). (**g**) One-way COX regression analysis of clinical and immunological characteristics and COVID-19 disease severity.

To detect the trend of NKeff cells subset over time, flow cytometry analysis was conducted on PBMCs from two subjects, Patient A with alive and Patient C (deceased). We found that the percentage of NKeff cells subset increased over time in Patient A (from 79.7% to 88.2%) and declined in Patient C (from 69.2% to 63.0%). Moreover, one-way COX regression analysis further found that NKeff cells subset was a substantial predictor of COVID-19 disease severity (coefficient = −0.096, 95% confidence interval: −0.174 to −0.018, *P*=0.016) (Figure 6).

### IL-6 and high glucose impair the activity and function of NK cells

Given the observed correlation between IL-6 and NK cell subsets, we conducted direct co-culture experiments using primary NK cells with IL-6 to investigate its biological effects. Our results demonstrated that IL-6 exposure significantly promoted the expansion of CD56^dim^ CD16^-^ NK cells subset while suppressing NKeff cells subset (Supplementary Figure S6). Notably, the CD56^bright^ CD16^-^ subset remained unaffected by IL-6 treatment. To further characterize the functional consequences of IL-6 signaling, we examined the expression levels of GZMB, perforin, IL-6 and IFN-γ. As shown in Supplementary Figure S7, IL-6 exposure substantially reduced cytotoxic potential in NK cells, evidenced by decreased GZMB, perforin, and IFN-γ expression, while enhancing IL-6 production itself. Importantly, all these effects were completely reversed by TCZ treatment, a clinically approved IL-6 receptor blocker, confirming the specific role of IL-6 receptor signaling in mediating NK cell dysfunction.

To investigate hyperglycemia-induced functional alterations in NK cells, we utilized an *in vitro* model using NK cells cultured under standard glucose concentration level (5 mmol/L), moderately hyperglycemic (15 mmol/L), and severely hyperglycemic (25 mmol/L) glucose conditions. Flow cytometric analysis revealed no significant changes in the frequencies of CD56^bright^ CD16^−^, CD56^dim^ CD16^−^, or NKeff cells subsets across glucose concentrations (Supplementary Figure S8). Strikingly, exposure to 15 mmol/L glucose markedly impaired NK cell effector function, demonstrating significant reductions in GZMB, perforin, and IFN-γ production, alongside an increase in IL-6 expression compared with standard glucose concentration level controls (5 mmol/L). These effects were further exacerbated under 25 mmol/L glucose (Supplementary Figure S9). Together, these findings demonstrate that hyperglycemia dose-dependently suppresses NK cell cytotoxicity while amplifying IL-6 production, independent of subset frequency alterations.

**Figure 7:**
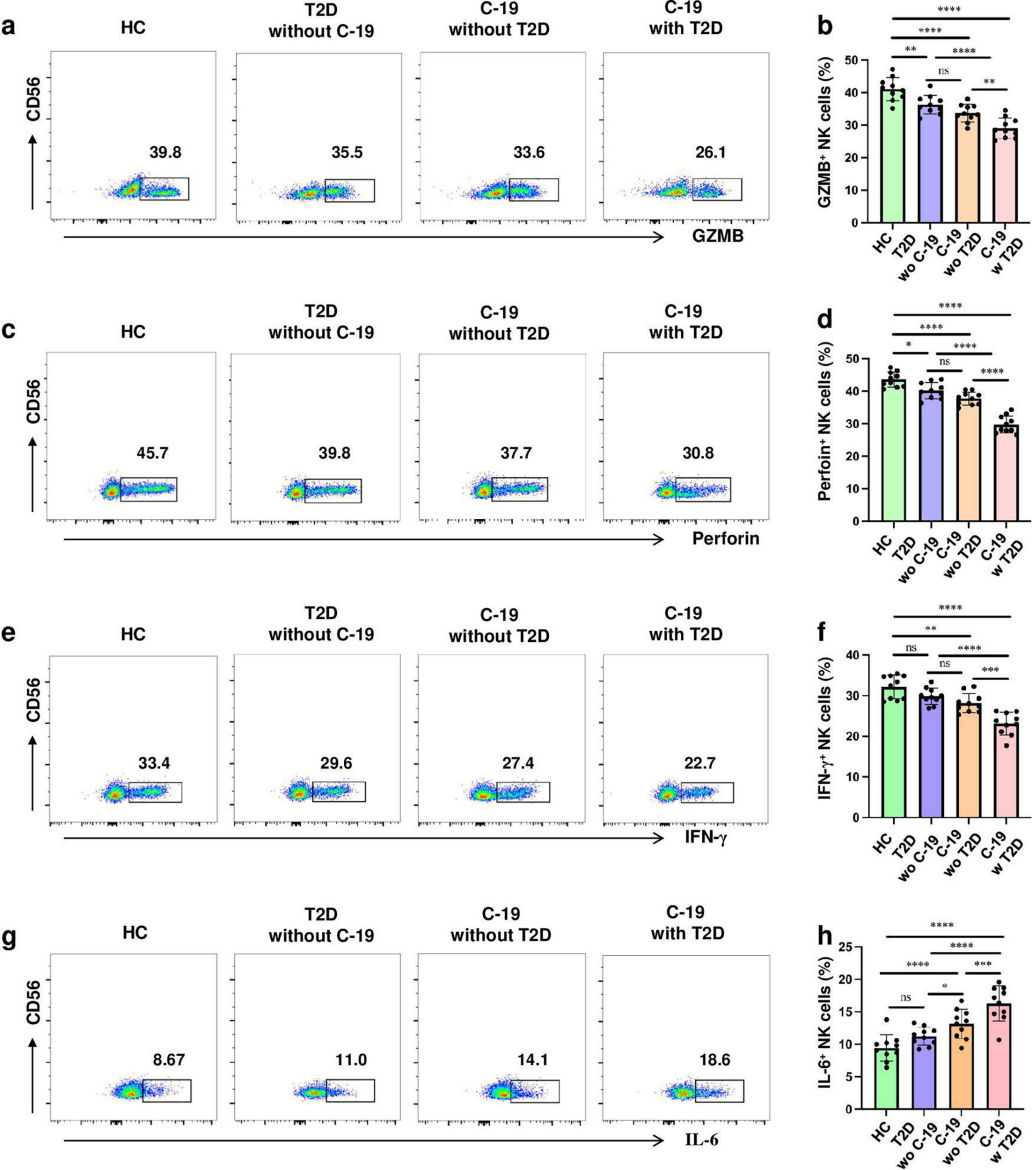
Function of NK cells undergoes alterations in COVID-19 patients comorbid with T2D. (**a, c, e, g**) Representative dot plots showing the expression of GZMB (**a**), perforin (**c**), INF-γ (**e**), IL-6 (**g**) by NK cells in HC, T2D without COVID-19, COVID-19 without T2D, and COVID-19 with T2D by flow cytometric analysis. (**b, d, f, h**) Statistical analysis of the expression of GZMB (**b**), perforin (**d**), INF-γ (**f**), IL-6 (**h**) by NK cells in HC, T2D, COVID-19 without T2D, and COVID-19 with T2D. Horizontal bars represent the mean ± SD. ns, not significant, *P < 0.05, **P < 0.01, ***P < 0.001, ****P < 0.0001 by one-way ANOVA followed by adjustments for multiple comparisons.

To comprehensively assess NK cell functional dynamics across clinical phenotypes, we performed comparative analyses of cytotoxic activity across four distinct cohorts: healthy controls, T2D without COVID-19, COVID-19 without T2D, and COVID-19 with T2D. Patients with comorbid COVID-19 and T2D exhibited significantly reduced expression of cytotoxic mediators (GZMB, perforin and IFN-γ) alongside elevated IL-6 levels compared with other groups, demonstrating a synergistic impairment of NK cell effector function in the dual-pathology cohort (Figure 7).

## Discussion

T2D, a prevalent metabolic disorder, is linked to persistent and low-level systemic inflammation [20]. COVID-19 patients have exhibited hyperinflammatory syndromes and cytokine storms [21]. NK cells, crucial elements of the innate immune response, have been connected to both T2D [22] and COVID-19 [23]. Despite this, the precise function of NK cells in individuals suffering from both COVID-19 and T2D remains to be fully elucidated. Our study presents novel findings that highlight the dysregulation of NK cell subsets and their immunophenotype in COVID-19 patients with comorbid T2D for the first time. We noted a considerable decrease in the NKeff cells subset among COVID-19 patients, marked by heightened KIR expression along with reduced levels of NKG2D and CD226. Additionally, the CD56^dim^ CD16^-^ cells subset, a relatively less understood non-classical group, exhibited a notable increase in COVID-19 patients. These effects were markedly pronounced among diabetic patients who were also COVID-19 positive. Furthermore, our data suggest that the NKeff cells subset could potentially act as a biomarker to forecast the severity of COVID-19.

NK cells are crucial to early protection against viruses and modulate the adaptive immune responses, both cellular and humoral. It was marked by the absence of CD3 and the expression of CD56. Prior research has indicated that the proportion of NK cells within the lymphocyte population of COVID-19 patients does not significantly deviate from that of healthy subjects, even across varying degrees of disease severity [23,24]. Our research further confirms this finding and expands upon it, demonstrating that this similarity in NK cell frequency persists across varying states of glucose metabolism, including those with comorbid dysglycemia.

Prior research has revealed a contraction of the NKeff cells subset, and no change in the CD56^bright^ CD16^-^ cells subset in individuals experiencing COVID-19, regardless of the severity of the disease [23]. In contrast, our study revealed a different relationship between disease severity and the prevalence of certain NK cell subsets among COVID-19 patients: the frequency of NKeff cells subset exhibited a decline, with a more significant trend in patients with T2D. Thus, our research reveals that the effect of COVID-19 on NK cells may be exacerbated in patients with comorbid T2D, suggesting that hyperglycemia may hasten the abnormal distribution and dysfunction of NK cells subsets. The cytotoxic capacity of NK cells derived from mice with T2D was found to be lower than that of NK cells derived from healthy mice [25]. Similarly, COVID-19 patients have poor cytotoxic function of NK cells upon stimulation [12]. Although a robust bidirectional relationship has been identified between T2D and COVID-19 [26], the operational mechanism of NK cells under the dual influence of T2D and COVID-19 remains unclear and requires further investigation. Numerous studies have demonstrated the unconventional CD56^dim^ CD16^-^ cells subset is present in the peripheral blood of healthy controls [27]. Recently, the increase in the CD56^dim^ CD16^-^ cells subset has been documented in various clinical scenarios, such as COVID-19, infectious mononucleosis, and malignancies [23,28,29]. Notably, our study revealed a marked expansion of the CD56^dim^CD16^-^ cells subset among COVID-19 patients who also had T2D, with the prevalence rising in line with disease severity. Previous studies found that the CD56^dim^ CD16^-^ cells subset is multifunctional cells and could be developed into other NK cells subsets [30,31]. However, the mechanism driving the expansion of the CD56^dim^ CD16^-^ cells subset remain to be elucidated. Further research is needed to clarify the role of CD56^dim^ CD16^-^ cells subset in the context of inflammation and hyperglycemia.

Glucocorticoids exert multifaceted immunomodulatory effects across both adaptive and innate immune systems. Mechanistically, these steroids have been shown to up-regulate PD-1 expression on NK cells, thereby suppressing their cytotoxic activity and functional responsiveness. Clinically, glucocorticoid administration in giant cell arteritis and polymyalgia rheumatica patients has been associated with quantitative depletion of circulating NK cell populations [32]. However, our study revealed preserved frequencies of total NK cells and functionally defined subsets despite therapeutic interventions. This apparent discrepancy with established literature may stem from pathophysiological differences between acute inflammatory conditions and COVID-19-associated immune dysregulation, dose-dependent effects of glucocorticoids (subtherapeutic vs. immunosuppressive regimens), and compensatory mechanisms in chronic T2D-COVID-19 comorbidity. Resolution of these conflicting observations warrants future investigations with expanded cohort sizes and longitudinal pharmacodynamic assessments. Additionally, emerging evidence suggests that preserved NK cell functionality and numerical integrity may constitute a critical determinant of successful antiviral therapeutic outcomes [33]. Our systematic evaluation demonstrated no significant modulatory effect of antiviral regimens on total NK cell frequencies or subset distributions. This observed stability in NK cell homeostasis during treatment suggests either target specificity of current antiviral agents sparing innate immune effectors or temporal dissociation between virological control and immunological normalization. Notably, while quantitative metrics remained stable, functional assays (e.g., IFN-γ production, cytotoxic granule mobilization) may be required to fully assess treatment-related immunomodulation.

The activity of NK cells is finely tuned by the balance of signals from both activating and inhibitory receptors [34]. An intriguing observation is that in COVID-19 patients with T2D, the NKeff cells subset exhibits an increased presence of inhibitory receptors, specifically KIRs, and a reduced presence of activating receptors DNAM1 (CD226) and NKG2D, as opposed to patients with only COVID-19 or T2D. A previous study has noted a heightened expression of inhibitory receptors coupled with a diminished expression of activating receptors in both NK cells and the NKeff cells subset among COVID-19 patients [23,24,35]. Within the scope of T2D, NKG2D expression was observed to decline, while KIR expression remained relatively stable compared with healthy blood donors [36]. These collective data suggest that elevated inflammatory and glycemic states may significantly perturb NK cell function. Our findings suggest that the dysregulation of NK cells subsets may be exacerbated under conditions of concurrent inflammation and hyperglycemia, potentially elucidating the exacerbated outcomes observed in COVID-19 patients with T2D [37]. Further experimental investigation is necessary to delineate the underlying mechanisms. Moreover, the identification of NK subsets expressing DNAM1 as pivotal to expedited recovery from SARS-CoV-2 infection [38] opens avenues for novel therapeutic strategies in COVID-19 management, particularly pertinent when the disease is complicated by T2D.

Patients with COVID-19 who also suffer from hyperglycemia exhibit significantly elevated levels of inflammatory markers [39]. A link has been identified between high levels of the pro-inflammatory cytokine IL-6 in serum and adverse outcomes for COVID-19 patients [40]. Our study suggests that individuals with both COVID-19 and T2D displayed increased IL-6 levels relative to individuals with COVID-19 only. IL-6, a multifunctional cytokine, is recognized as a pivotal component of the immune system, bridging innate and adaptive immunity, as well as autoimmunity [41]. IL-6 has been implicated in directly reducing the cytotoxic capabilities of NK cells and may also down-regulate the expression of NKG2D to reduce the efficacy of NK cell activity [42,43]. Our *in vitro* experiment revealed that IL-6 and high glucose levels inhibited the function of NK cells by reducing the expression of GZMB, perforin, and IFN-γ, while simultaneously increasing the expression of IL-6. The identified IL-6/glucose-NK cell axis may explain the exacerbated severity of COVID-19 in patients with diabetes, where pre-existing metabolic stress (chronic hyperglycemia) and acute viral-induced inflammation create a 'double-hit' scenario on antiviral immunity. Furthermore, a randomized clinical trial revealed that tocilizumab, a monoclonal antibody targeting the IL-6 receptor, significantly reduced the probability of severe progression to mechanical ventilation or death in patients hospitalized with COVID-19 pneumonia [44]. Therefore, therapeutic interventions targeting the overexpression of IL-6 and glucose metabolism may serve as a promising strategy to bolster the immune response against viruses mediated by NK cells, particularly in cases where COVID-19 is comorbid with T2D.

A recent meta-analysis indicated that elevated fasting blood glucose levels upon hospital admission are correlated with an unfavorable prognosis among COVID-19 patients [45]. We found that the frequency of NKeff cells subset may be an indicator for COVID-19 disease severity, with a lower frequency correlating with poorer outcomes. Our findings underscore the significance of the NKeff cells subset in combating viral infection. The abnormal glucose metabolism and overwhelming inflammation may both accelerate the progress of COVID-19 disease, indicating that future healthcare guidelines should not only address the management of COVID-19 but also integrate strategies for mitigating hyperglycemia to prevent preexisting health inequalities being further exacerbated. Utilization of Continuous Glucose Monitoring (CGM) was found to link to better glycemic control in T2D patients compared with traditional self-monitoring of blood glucose, represented by increased time in range of 6.36% (1 hour and 32 minutes per day) [46]. Thus, patients with COVID-19 complicated by T2D may achieve better blood glucose control, improved inflammation, and better health outcomes by wearing a CGM.

However, there are some limitations in this study. First, this study lacked detailed mechanistic insight. Second, the small cohort size (particularly the analysis of the association between the NKeff cells subset and COVID-19 severity in only two cases) may restrict the broader applicability of our findings and requires validation through longitudinal studies. Third, this retrospective nature of the design may constrain the extent to which we can infer causality or delineate the dynamic changes in NK cell behavior amidst the complex interplay between COVID-19 and T2D. Future research should prioritize 1) mechanistic studies clarifying IL-6/hyperglycemia-induced NK cell dysfunction through combined *in vivo* (e.g., IL-6 transgenic models) and *in vitro* approaches; 2) systematic characterization of NK cell immunometabolic crosstalk with T cells/monocytes in patient samples and tissue-specific dynamics in viral target organs via spatial multi-omics; 3) multicenter prospective cohorts with sufficient sample size and longitudinal designs to establish temporal relationships between NK cell activity and COVID-19/T2D progression. These approaches will enhance biological insight and therapeutic translation while addressing current limitations in generalizability. Despite these limitations, the strength of this study lies in its depiction of the early immune changes in NK cell subsets in patients with T2D during the initial infection of COVID-19 for the first time, enabling us to explore potential pathophysiology and understand early immune modulation. This insight underscores the imperative to incorporate considerations of NK cells dynamics and inflammatory processes in both the clinical management and research endeavors focused on T2D.

In conclusion, our study revealed alterations in NK cells subsets in COVID-19 patients, with exacerbated dysregulation in those with T2D. CD56^dim^CD16^+^ cell subsets may serve as indicators of the severity of COVID-19 disease.

**Clinical Perspectives**

- Type 2 Diabetes (T2D) is essentially a chronic, low-grade inflammatory disease, with immune dysregulation playing an important role in the occurrence and progression of T2D and its related metabolic disorders and complications. Natural killer (NK) cells serve as the frontline defenders against tumor cells and play a critical role in immune surveillance against viral infections. However, the role of NK cells in the context of both hyperglycemia and the presence of SARS-CoV-2 remains unclear.
- In this study, using flow cytometry of peripheral immune cells from patients with COVID-19, we found the dysregulation of NK cells subsets, with increased CD56^dim^ CD16^-^ cells subset and decreased CD56^dim^ CD16^+^ cells subset. Importantly, patients with severe COVID-19 showed a higher prevalence of CD56^dim^CD16^-^ cells subset and a lower prevalence of CD56^dim^ CD16^+^ cells subset, with these trends being even more pronounced in those with comorbid T2D. Additionally, the CD56^dim^ CD16^+^ cells subset may serve as an indicator of the severity of COVID-19 disease.
- Our study identified a significant deep shift in the immune profile of NK cells among COVID-19 patients with T2D, which may represent a critical factor influencing the clinical outcomes in these patients.

## Supplementary material

Online supplementary figure 1

Online supplementary figure 2

Online supplementary figure 3

Online supplementary figure 4

Online supplementary figure 5

Online supplementary figure 6

Online supplementary figure 7

Online supplementary figure 8

Online supplementary figure 9

Online supplementary material

## Data Availability

Data are available directly from the corresponding author upon reasonable written request.

## Open Access

This article has been published open access under our Subscribe to Open programme, made possible through the support of our subscribing institutions, learn more here: https://portlandpress.com/pages/open_access_options_and_prices#conditional

## Abbreviations

BMI: body mass index
GZMB: granzyme B
IFN-γ: Interferon-γ
IL-6: interleukin-6
NK: natural killer
NKeff cells: effector NK cells
PBMCs: peripheral blood mononuclear cells
TCZ: tocilizumab
T2D: type 2 diabetes
