# Supplementary figures and images for: Dysregulation of NK cell subsets and phenotypes in COVID-19 patients with comorbid type 2 diabetes
Source: Clin Sci (Lond). 2025 Jun 23;139(12):683–702. doi: 10.1042/CS20243133 (PMC12238817; doi:10.1042/CS20243133)

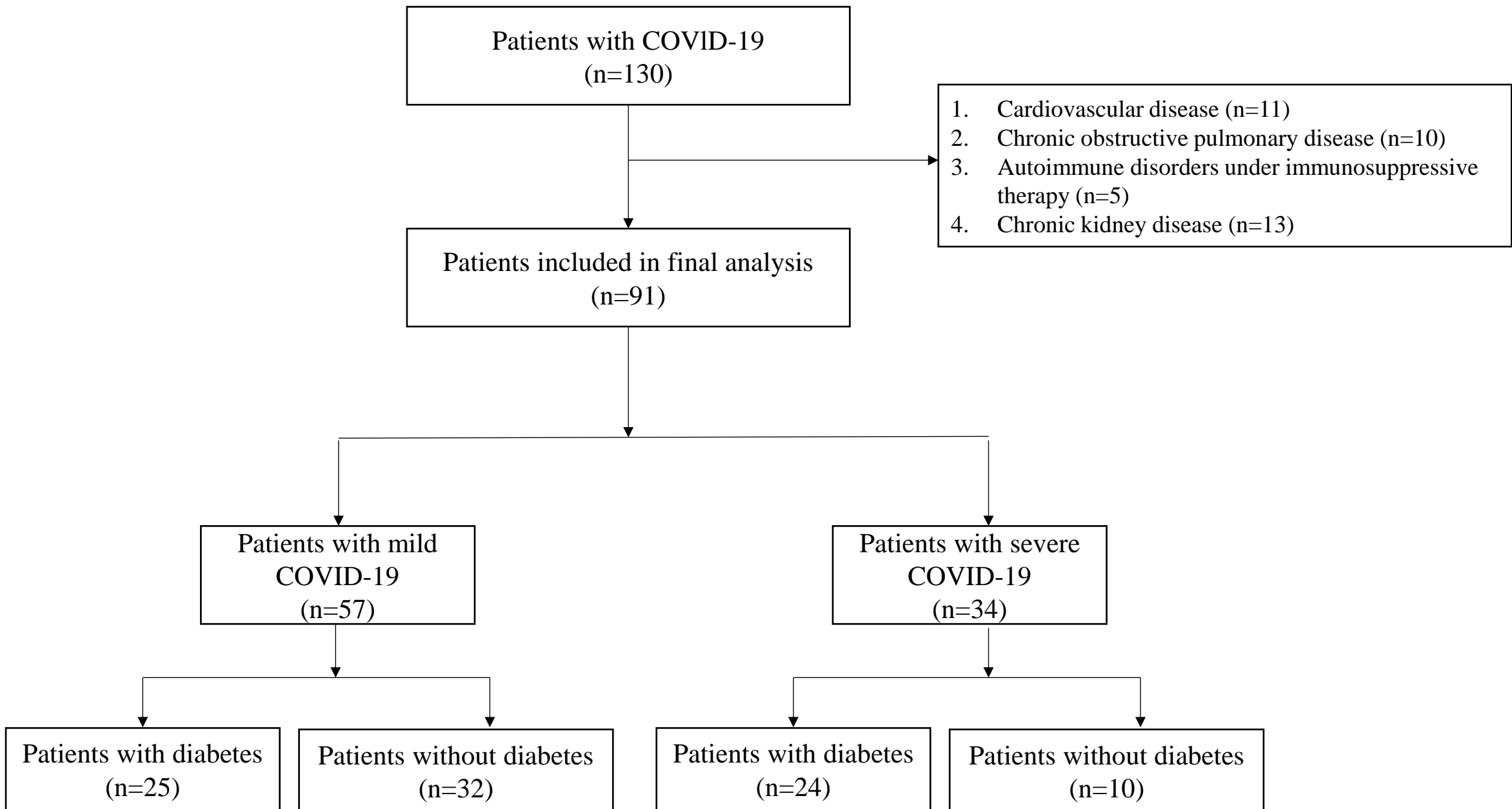

Supplement: Online supplementary figure 1 [file cs-139-12-CS20243133-supp1.pdf]

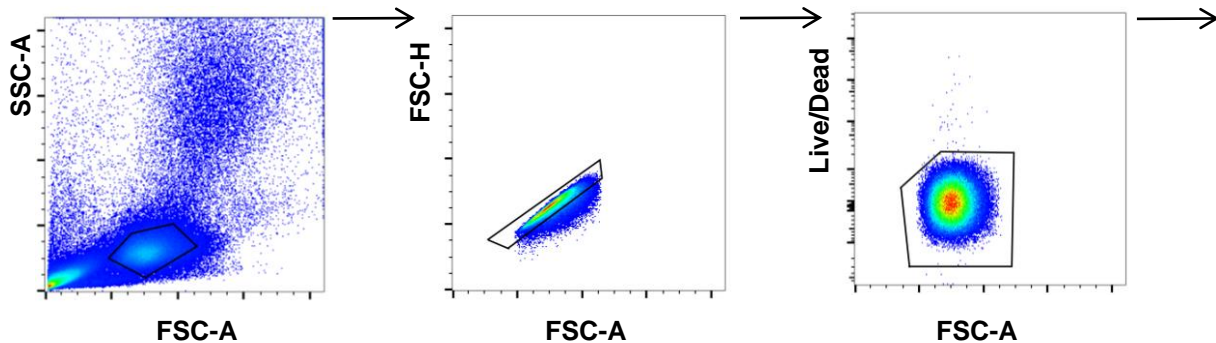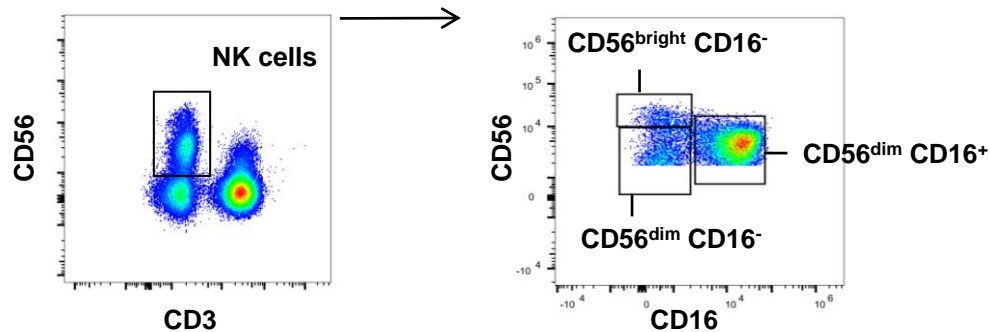

Supplement: Online supplementary figure 2 [file cs-139-12-CS20243133-supp2.pdf]

**a**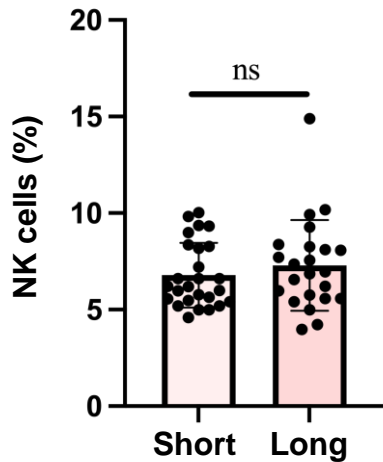**b**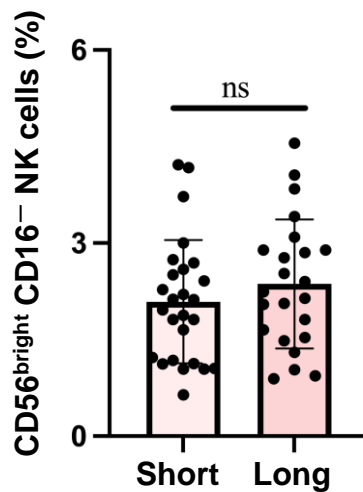**c**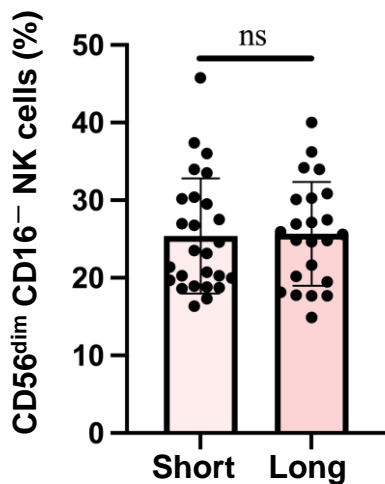**d**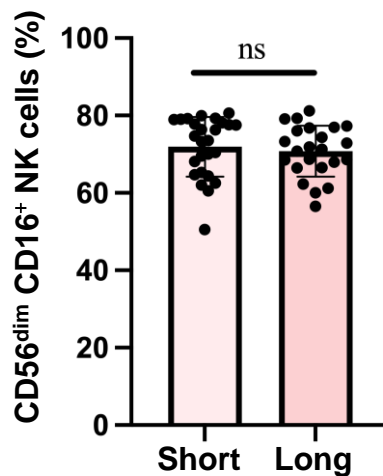

Supplement: Online supplementary figure 3 [file cs-139-12-CS20243133-supp3.pdf]

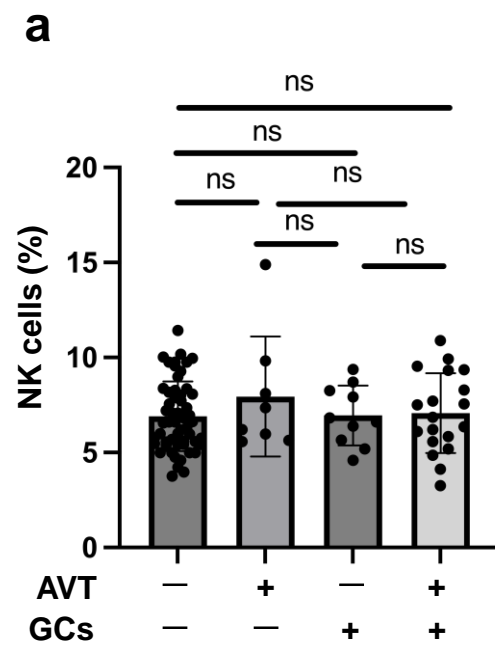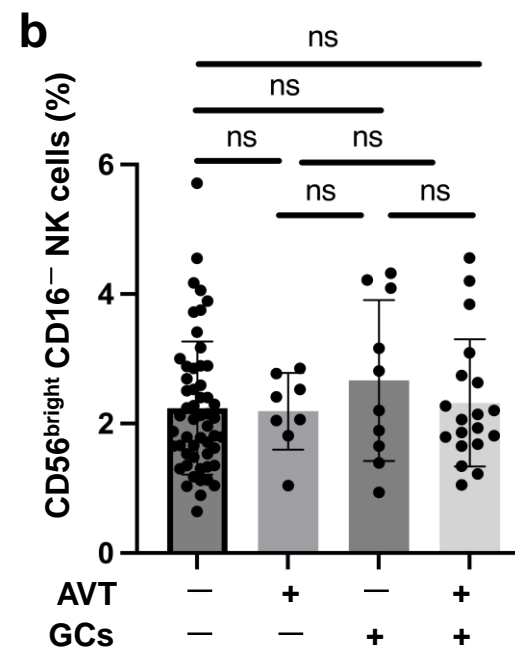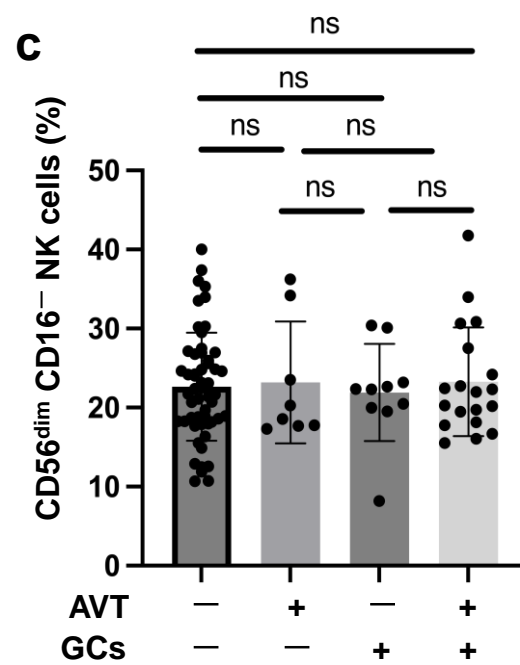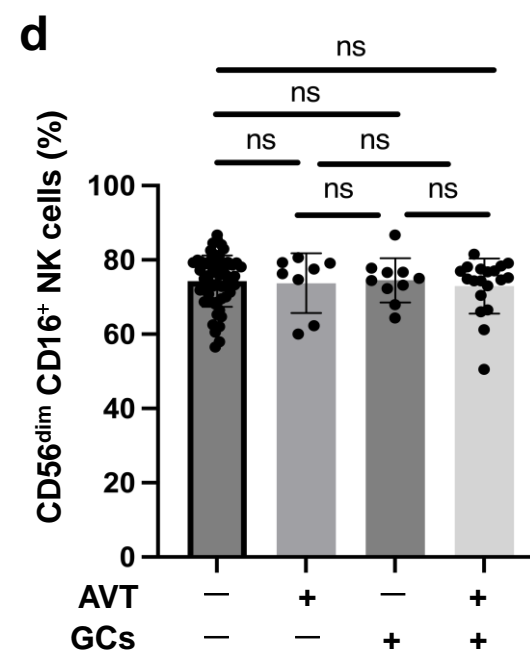

Supplement: Online supplementary figure 4 [file cs-139-12-CS20243133-supp4.pdf]

Gated on CD56<sup>Bright</sup> CD16<sup>-</sup> NK cells

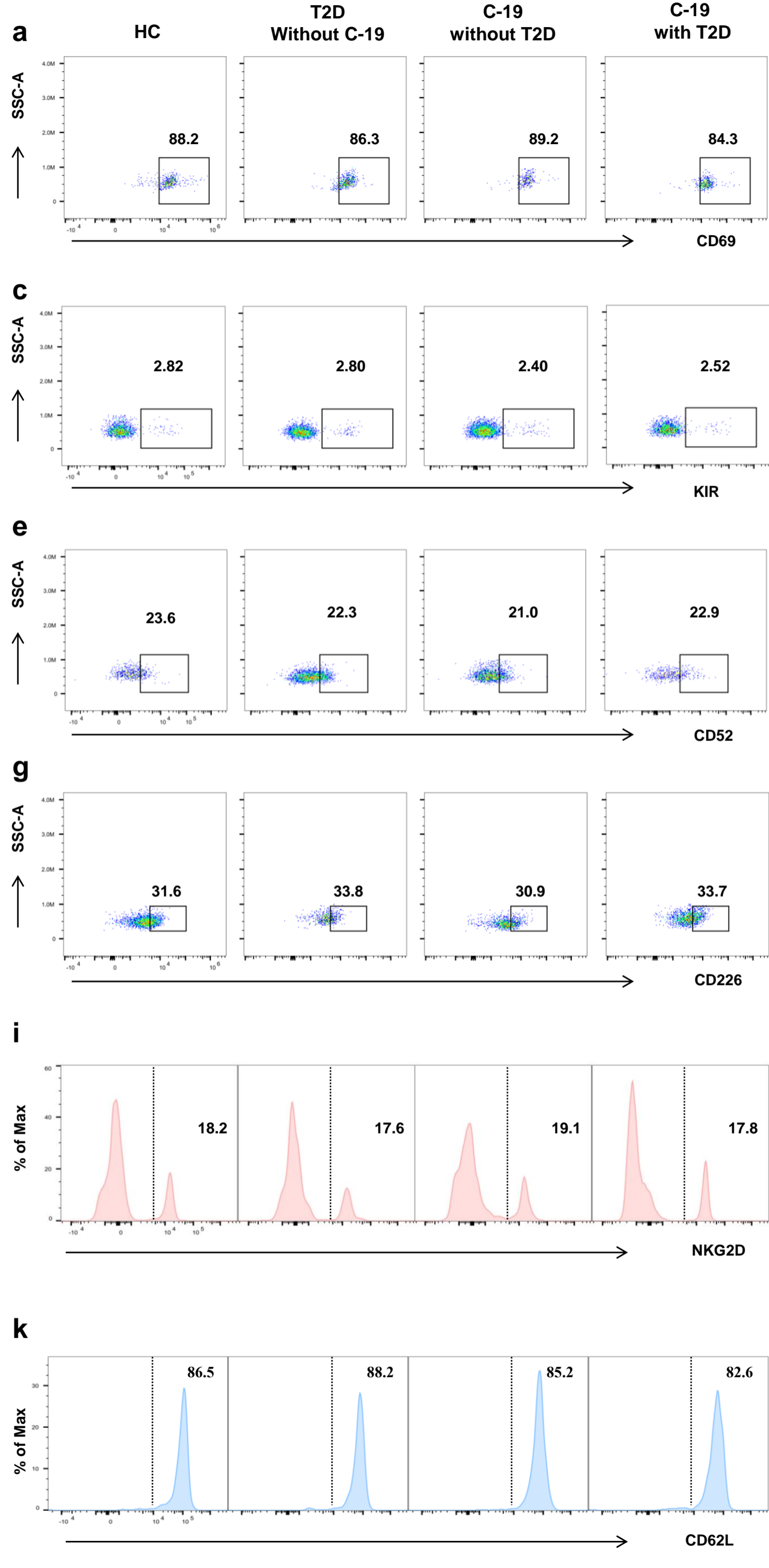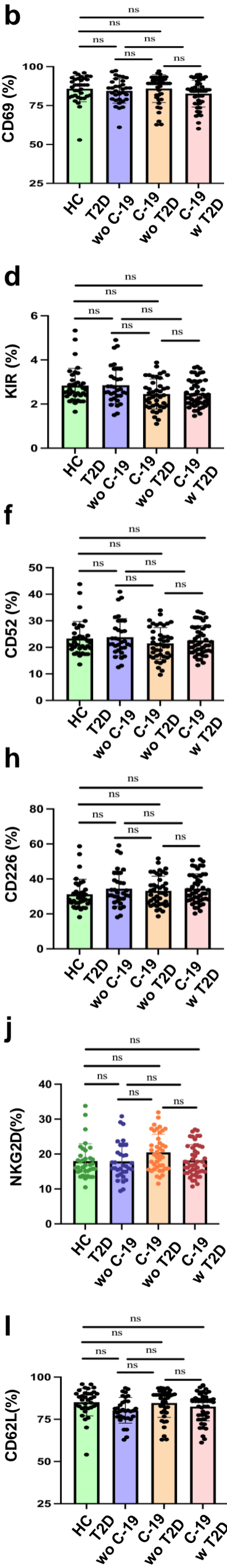

Supplement: Online supplementary figure 5 [file cs-139-12-CS20243133-supp5.pdf]

**a**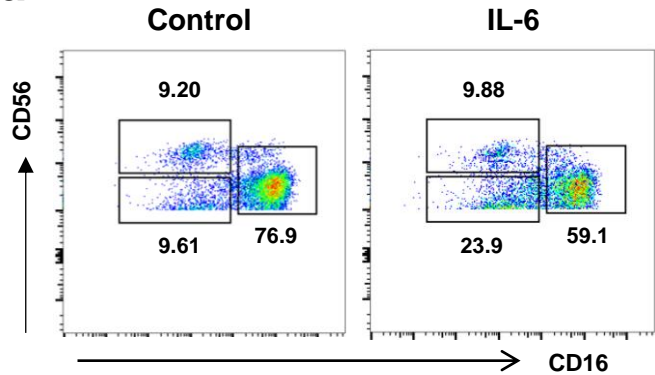**b**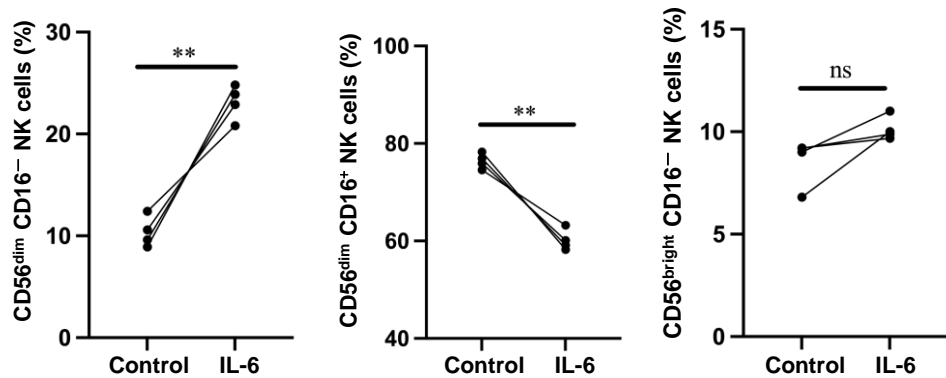

Supplement: Online supplementary figure 6 [file cs-139-12-CS20243133-supp6.pdf]

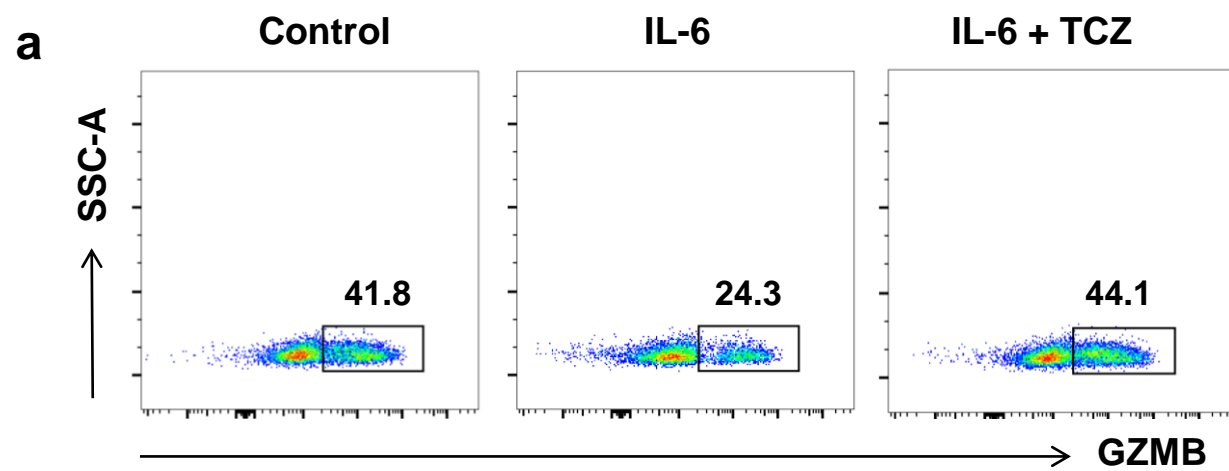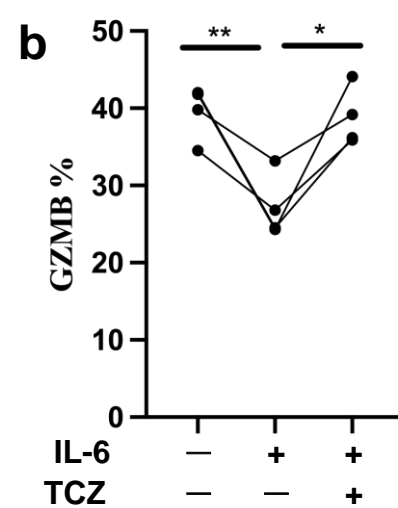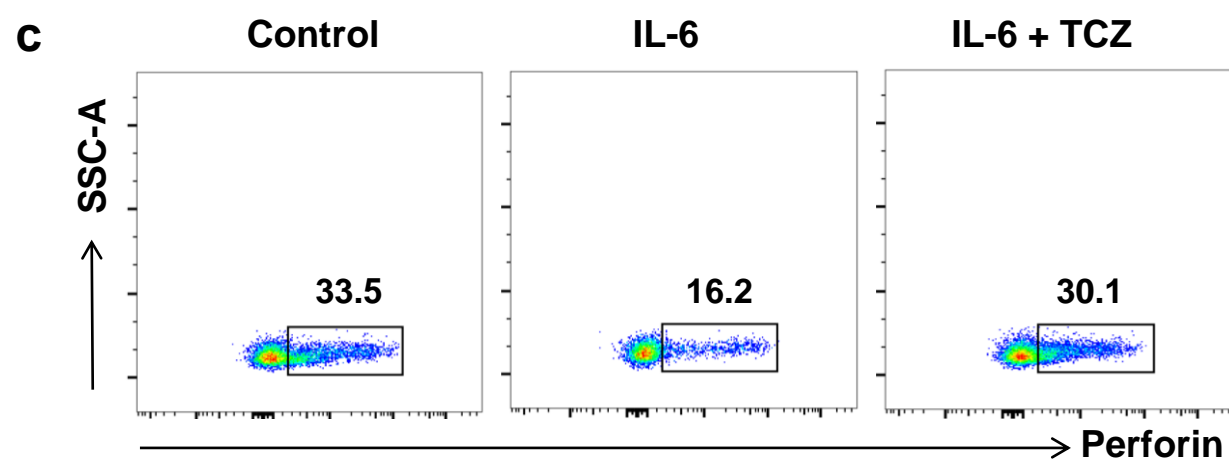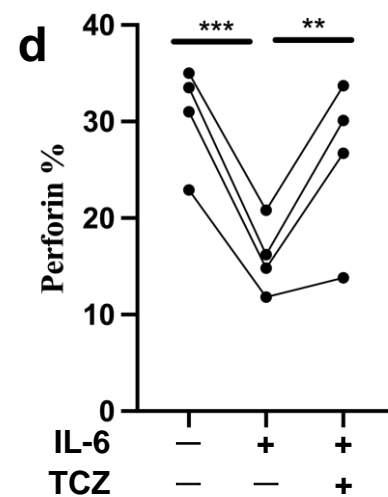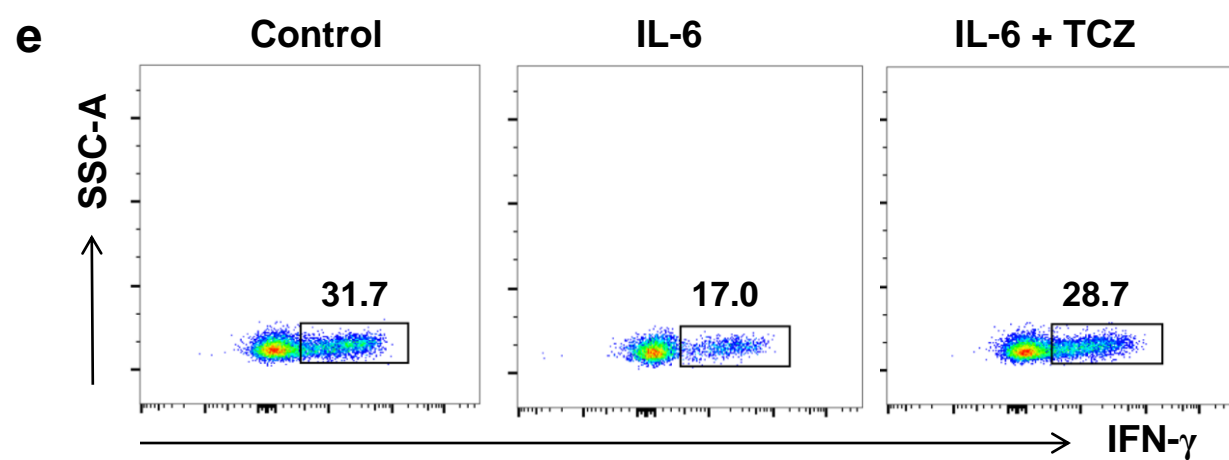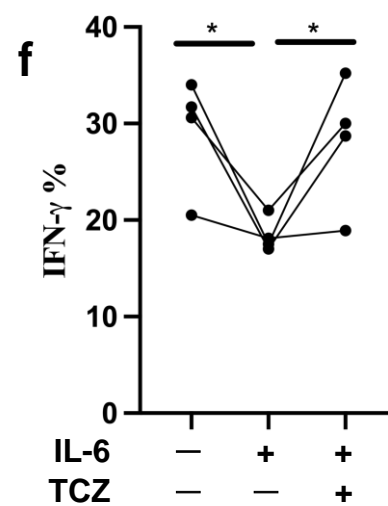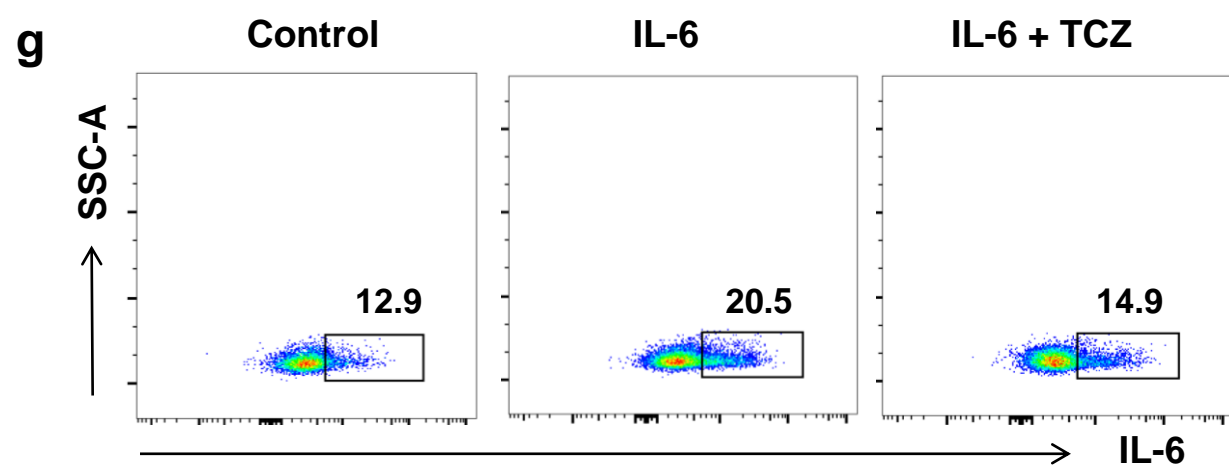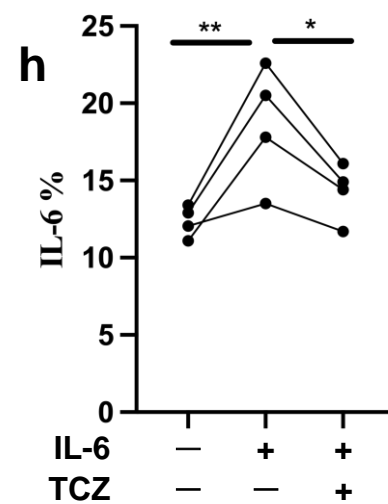

Supplement: Online supplementary figure 7 [file cs-139-12-CS20243133-supp7.pdf]

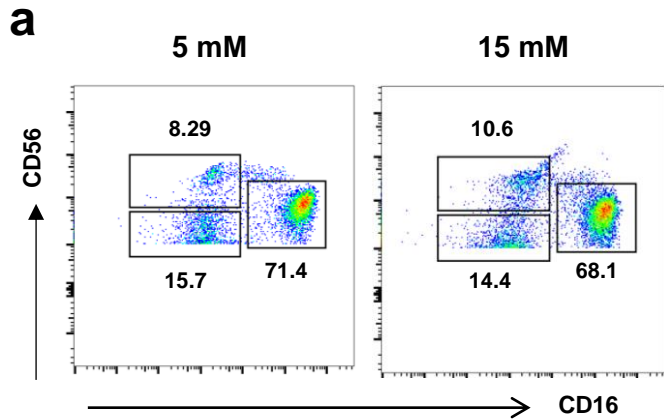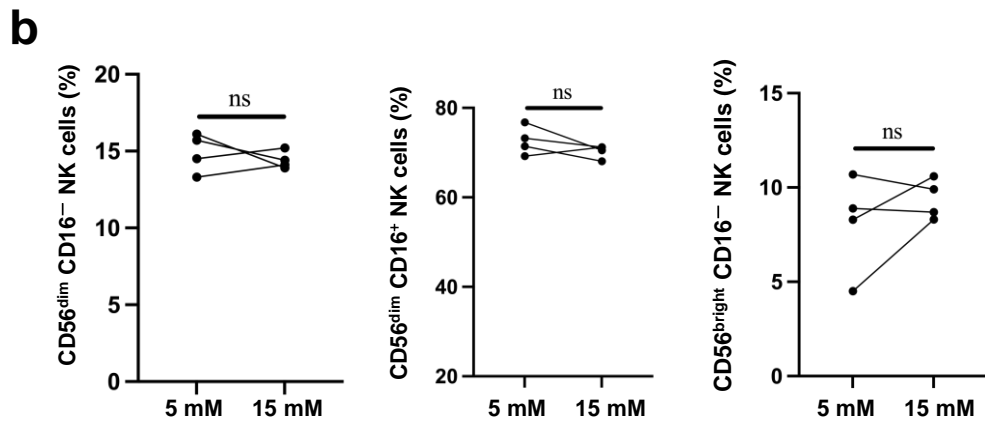

Supplement: Online supplementary figure 8 [file cs-139-12-CS20243133-supp8.pdf]

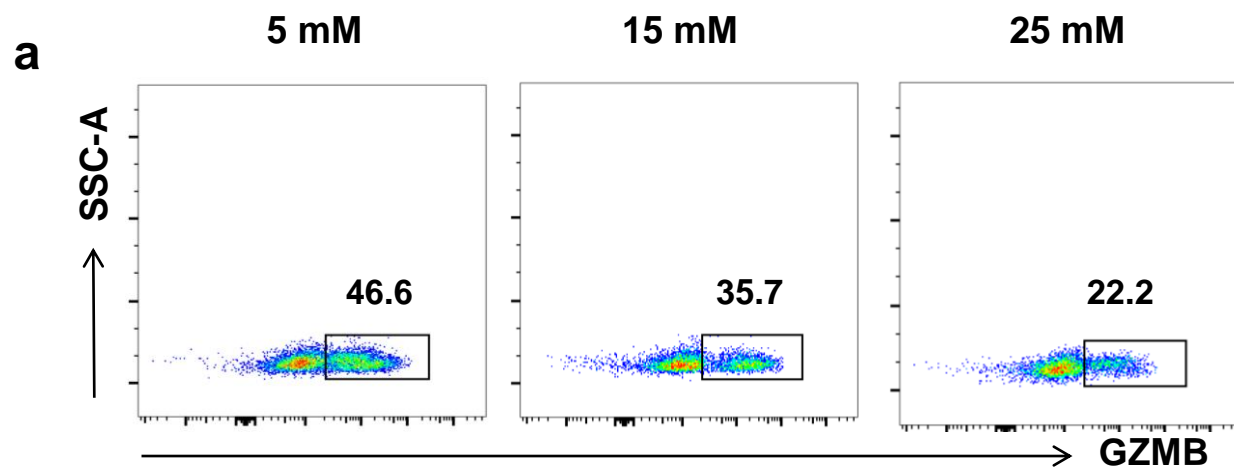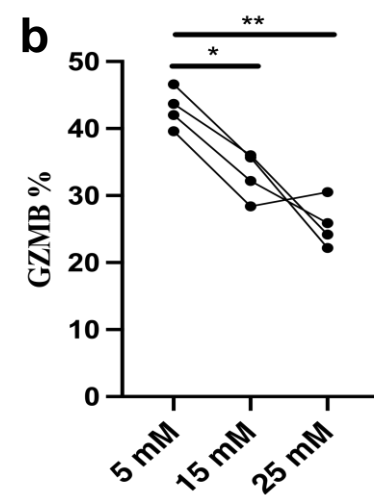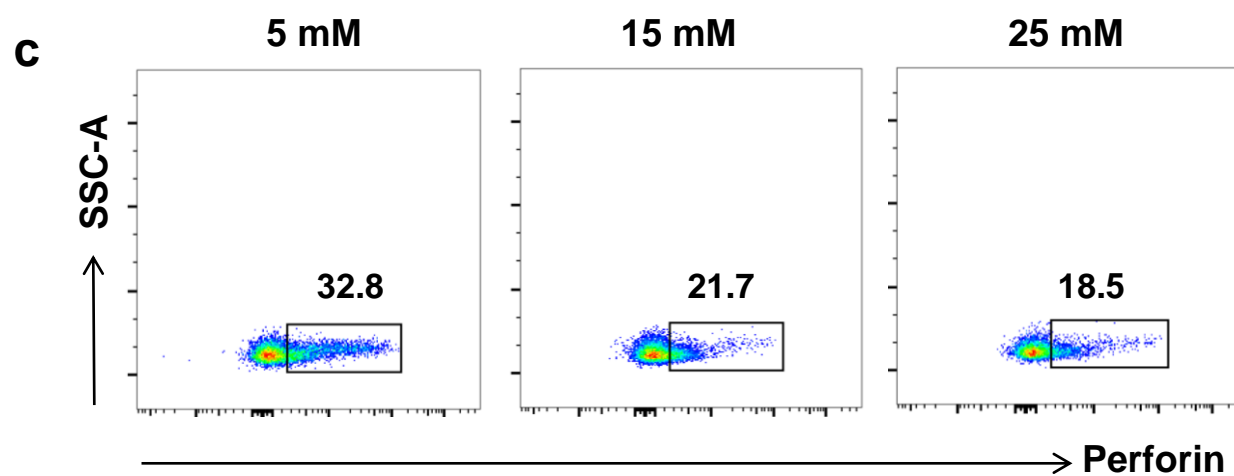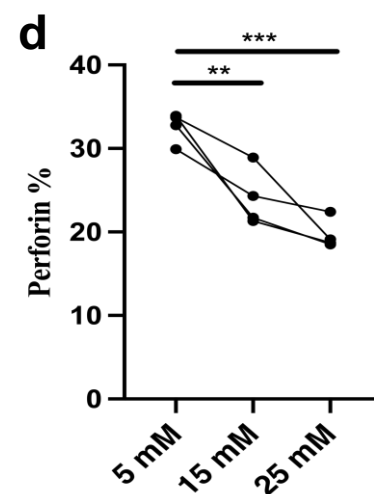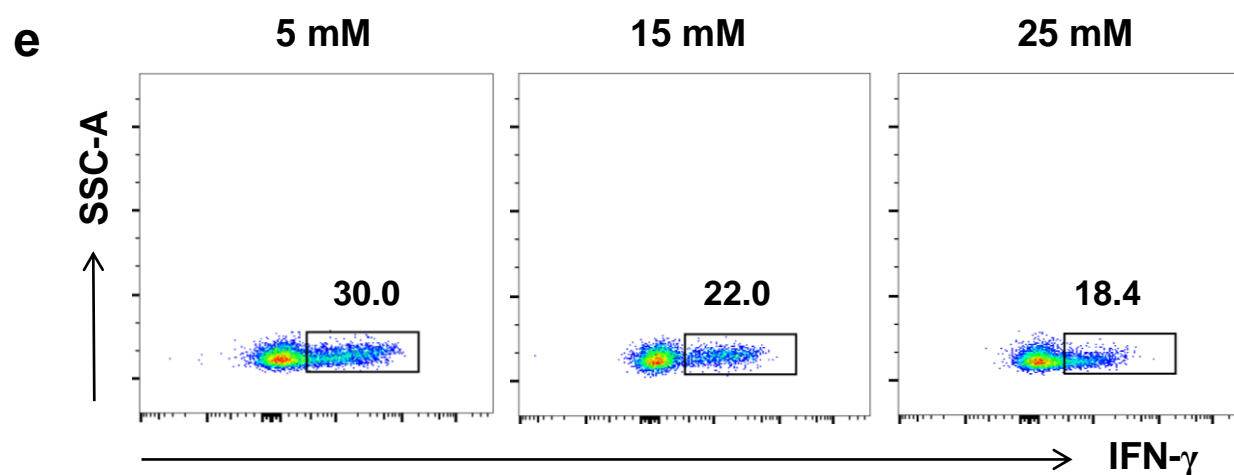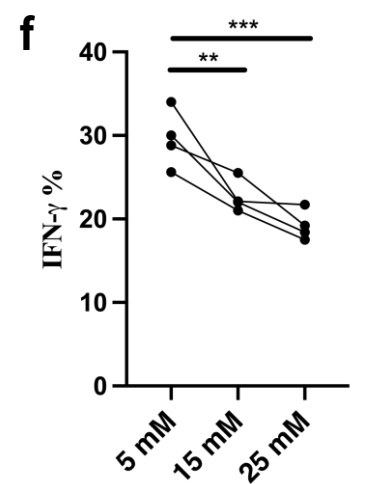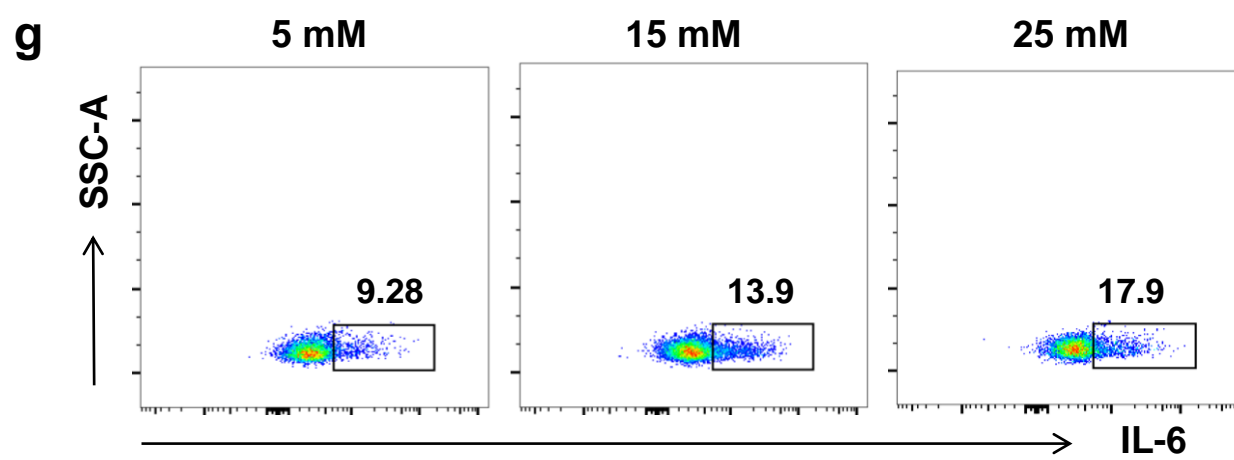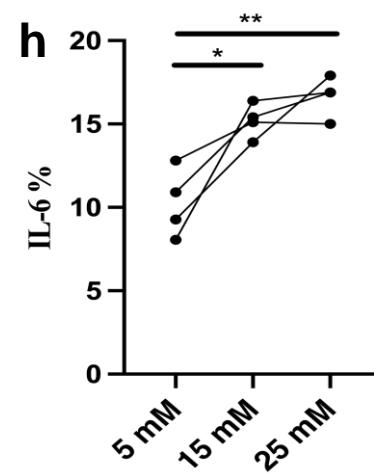

Supplement: Online supplementary figure 9 [file cs-139-12-CS20243133-supp9.pdf]
