# Supplementary material for: Dysregulation of NK cell subsets and phenotypes in COVID-19 patients with comorbid type 2 diabetes
Source: Clin Sci (Lond). 2025 Jun 23;139(12):683–702. doi: 10.1042/CS20243133 (PMC12238817; doi:10.1042/CS20243133)
Supplement: Online supplementary material [file cs-139-12-CS20243133-supp10.docx]

**Dysregulation of NK cell subsets and phenotypes in**

**COVID-19 patients with comorbid type 2 diabetes**


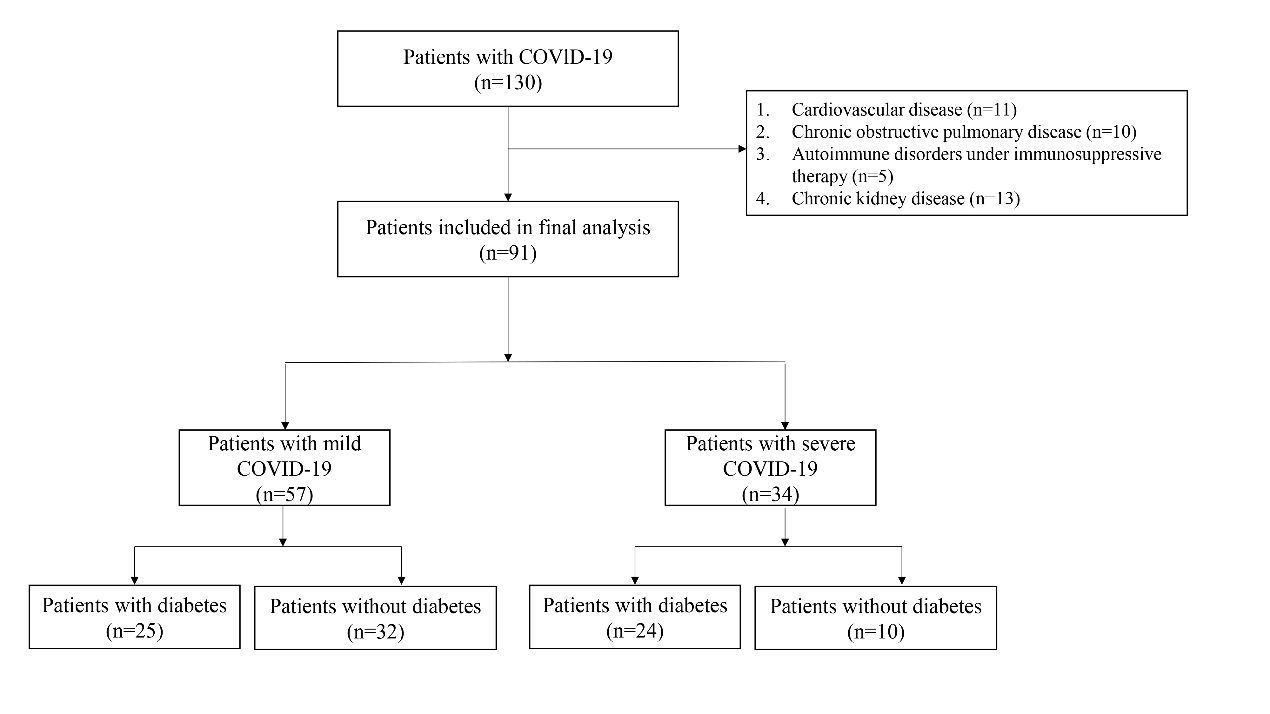


**Supplementary figure 1. Flowchart of patient enrollment and exclusion.**


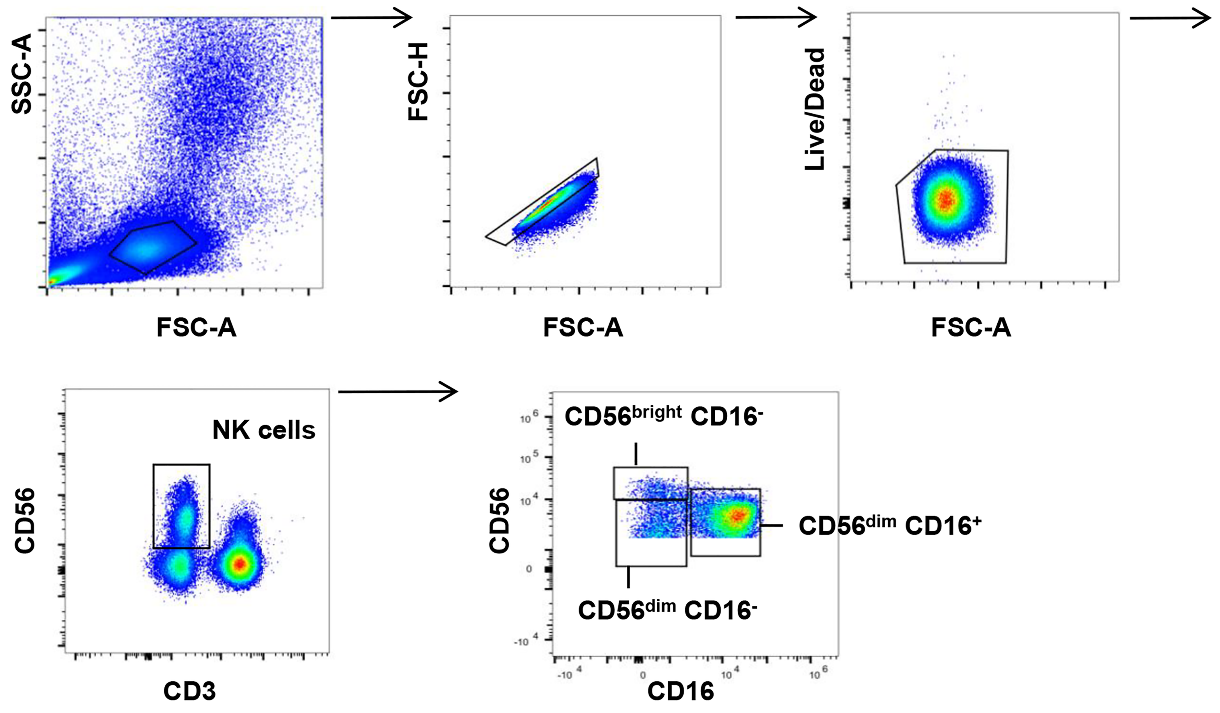


**Supplementary figure 2. Gating strategy and proportion of each cell population among NK cells.**


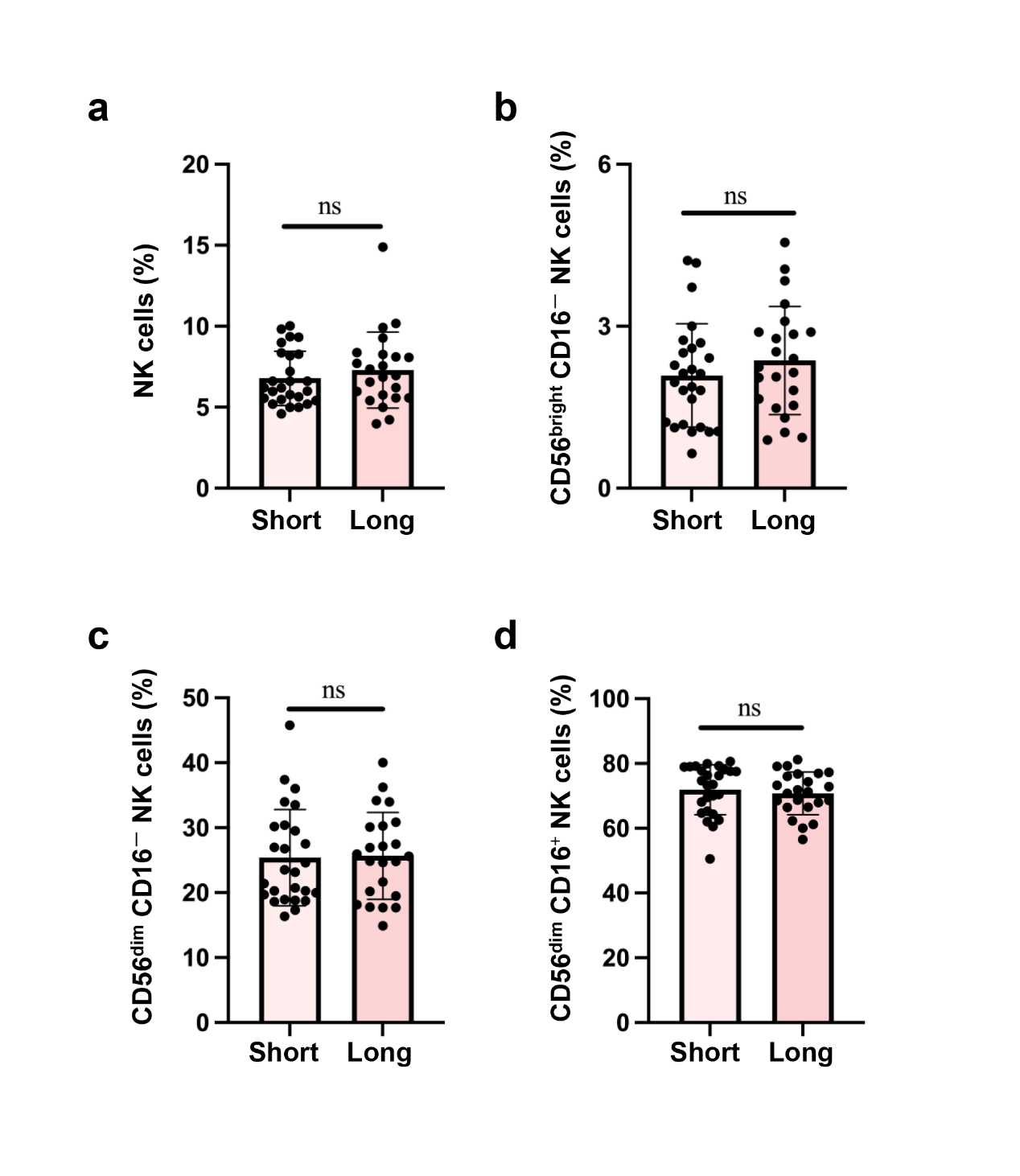


**Supplementary figure 3. Stratified analysis of total NK cells and functionally distinct subsets (CD56ᵇʳⁱᵍʰᵗCD16⁻, CD56ᵈⁱᵐCD16⁻, NKeff) in COVID-19 patients with T2D reveals duration-independent distribution patterns between short-term (≤5 years, n=26) and long-term (>5 years, n=23) disease subgroups.**

(a, b, c, d) Statistical analysis of the frequencies of total NK cells (a), CD56^bright^ CD16^-^ cells subset (b), CD56^dim^ CD16^-^ cells subset (c), and NKeff cells subset (d).

ns, not significant.


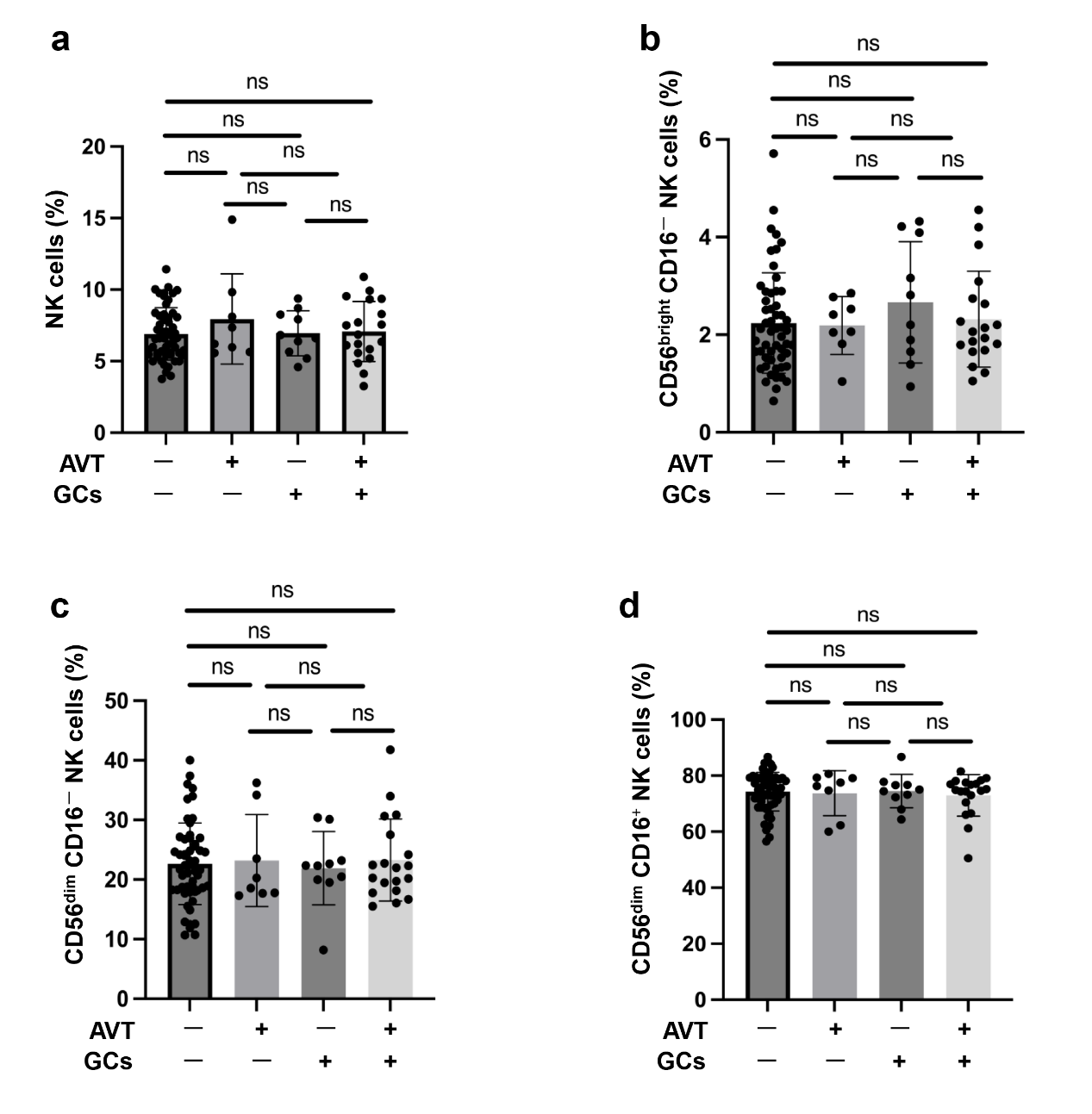


**Supplementary figure 4.** **Pharmacological modulation analysis of NK cell populations in patients with COVID-19.**

(a, b, c, d) Total NK cells (a), CD56ᵇʳⁱᵍʰᵗCD16⁻ cells subset (b), CD56ᵈⁱᵐCD16⁻ cells subset (c), and NKeff cells subset (d) frequencies in glucocorticoid-treated (GCs, n=10), antiviral-exposed (AVT, n=8), both glucocorticoid and antiviral-treated (n=19) and untreated control (n=54) groups.

ns, not significant.


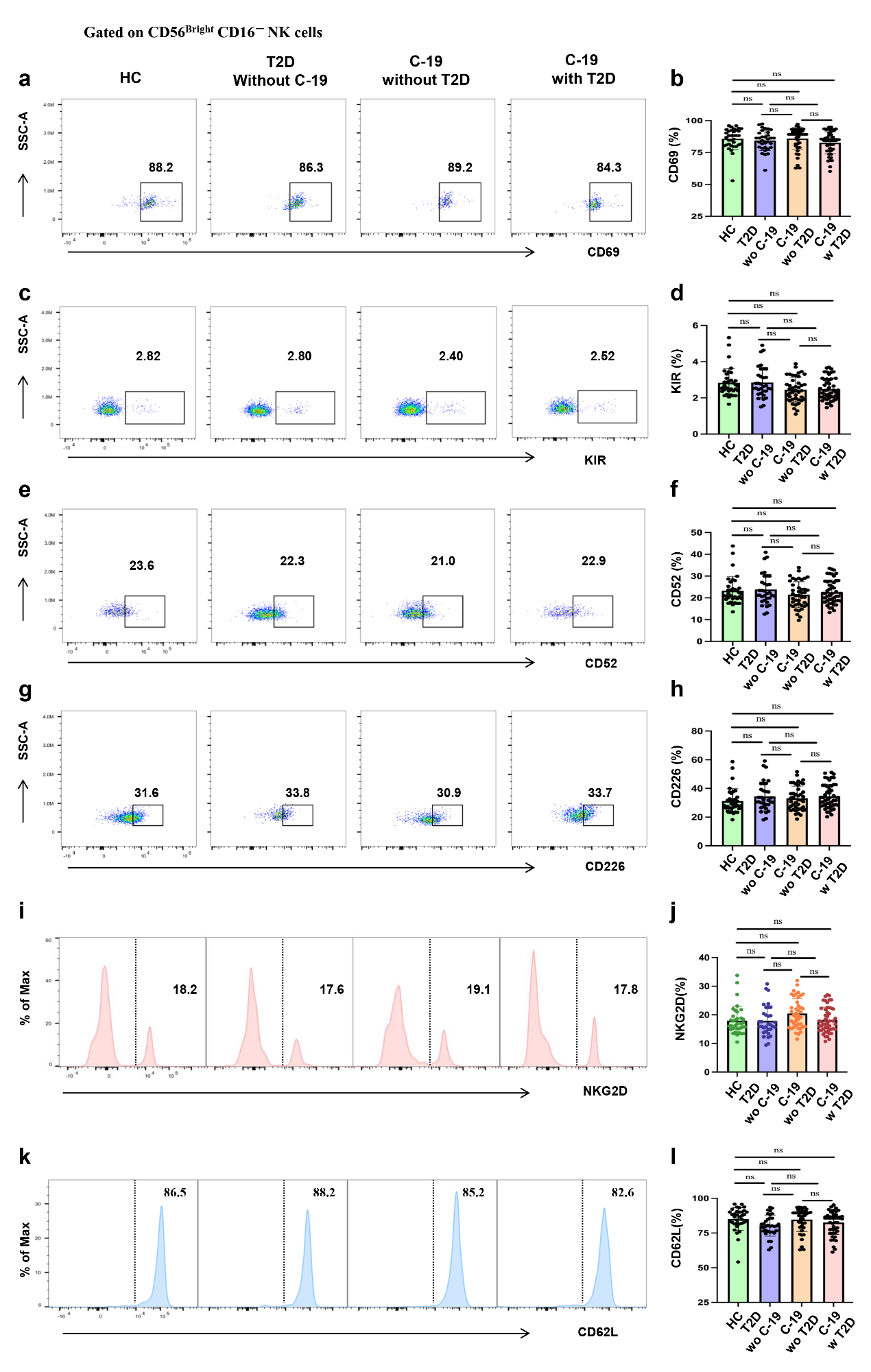


**Supplementary figure 5. The CD56^bright^ CD16^-^ cells subset immunophenotype did not fluctuate strongly in patients with COVID-19 combined with type 2 diabetes.**

(a, c, e, g, i, k) Representative dot plots showing the expression of CD69 (a), KIR (c), CD52 (e), CD226 (g), NKG2D (i) and CD62L (k) by CD56^bright^ CD16^-^ cells subset in HC, T2D without COVID-19, COVID-19 without T2D, and COVID-19 with T2D by flow cytometric analysis. (b, d, f, h, j, l) Statistical analysis of the expression of CD69 (b), KIR (d), CD52 (f), CD226 (h), NKG2D (j) and CD62L (l) by CD56^bright^ CD16^-^ cells subset in HC (n=35), T2D without COVID-19 (n=33), COVID-19 without T2D (n=42), and COVID-19 with T2D (n=49).

Horizontal bars represent the mean ± SD. ns, not significant by one-way ANOVA followed by adjustments for multiple comparisons.


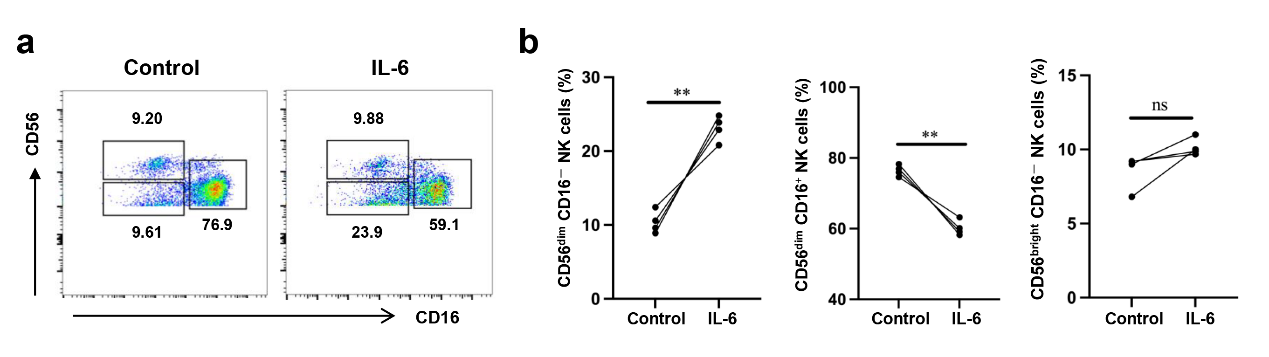


**Supplementary figure 6. Changes in the frequencies of NK cell subsets following co-culturing with IL-6.**

(a) Representative dot plots showing the frequency of CD56^bright^ CD16^-^ cells subset, CD56^dim^ CD16^-^ cells subset, NKeff cells subset in control and IL-6-treated PBMCs by flow cytometric analysis. (b) Statistical analysis of NK cells subsets in control and IL-6-treated PBMCs.

ns, not significant, ** *P* < 0.01.


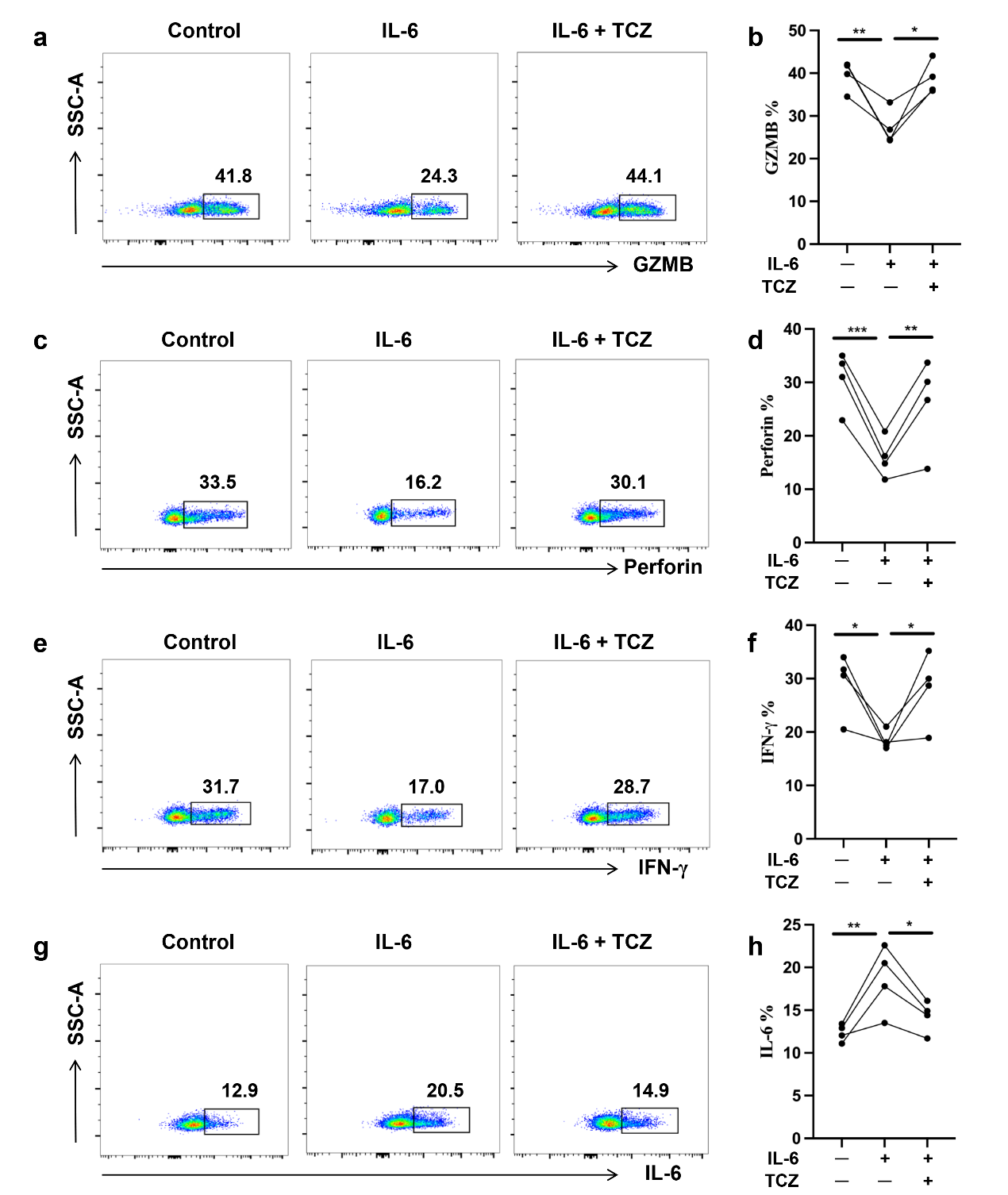


**Supplementary figure 7. Effect of IL-6 treatment on human NK cells in the presence or absence of tocilizumab (TCZ).**

(a, c, e, g) Representative dot plots showing the expression of GZMB (a), perforin (c), INF-γ (e), IL-6 (g) by total NK cells in healthy donors by flow cytometric analysis. (b, d, f, h) Statistical analysis of the expression of GZMB (b), perforin (d), INF-γ (f), IL-6 (h) by total NK cells in healthy donors (n=4).

**P* < 0.05, ***P* < 0.01, ****P* < 0.001.


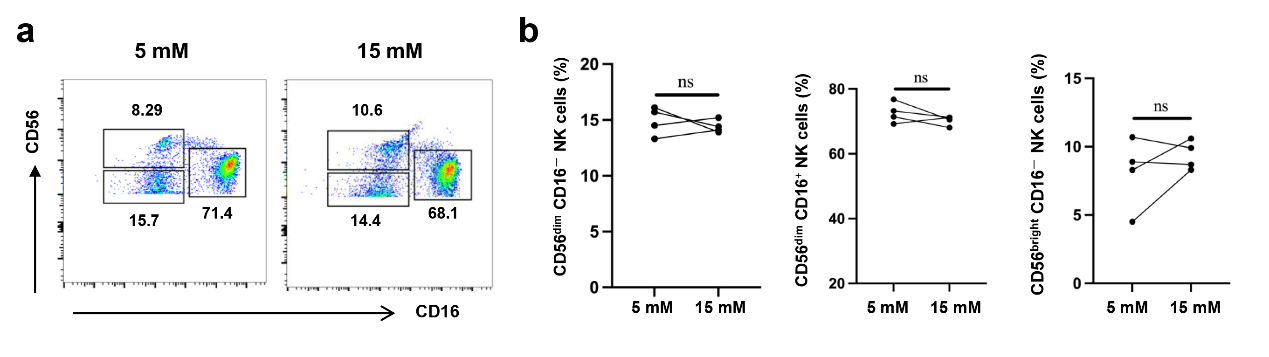


**Supplementary figure 8. Changes in the frequencies of NK cell subsets following co-culturing with different glucose concentrations.**

(a) Representative dot plots showing the frequency of CD56^bright^ CD16^-^ cells subset, CD56^dim^ CD16^-^ cells subset, NKeff cells subset after treatment with 5 mmol/L and 15 mmol/l glucose by flow cytometric analysis. (b) Statistical analysis of NK cells subsets after treatment with 5 mmol/L and 15 glucose mmol/L.

ns, not significant.


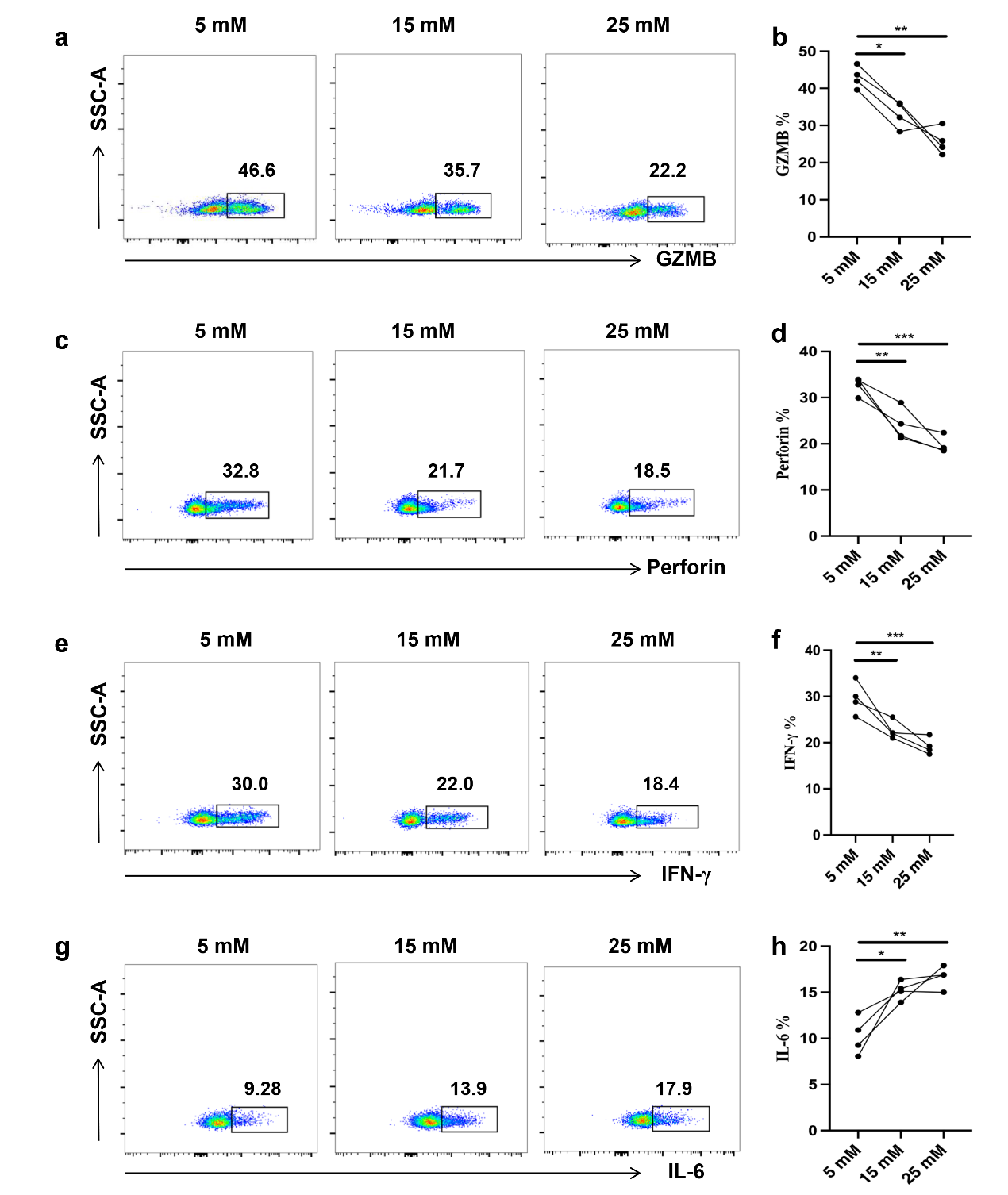


**Supplementary Figure 9. Effects of differential glucose concentrations on human NK cells (5 mmol/L, 15 mmol/L, and 25 mmol/L).**

(a, c, e, g) Representative dot plots showing the expression of GZMB (a), perforin (c), INF-γ (e), IL-6 (g) by total NK cells in healthy donors by flow cytometric analysis. (b, d, f, h) Statistical analysis of the expression of GZMB (b), perforin (d), INF-γ (f), IL-6 (h) by total NK cells in healthy donors (n=4).

**P* < 0.05, ***P* < 0.01, ****P* < 0.001.

**Supplementary Table 1. FCM Panel**

| ***Marker*** | ***Flurochorme*** | ***Clone*** | ***Vendor*** | ***Catalog*** |
| --- | --- | --- | --- | --- |
| **NK cell subsets** | | | | |
| CD3 | APC-Cy7 | SK7 | BD Pharmingen | 557832 |
| CD56 | PerCP-Cy5.5 | HCD56 | BioLegend | 318321 |
| CD16 | FITC | 3G8 | BioLegend | 302006 |
| Fixable Dye | Zombie Aqua^TM^ | / | BioLegend | 423101 |
| **NK cell subsets_phenotypes** | | | | |
| CD3 | BV510 | OKT3 | BioLegend | 317332 |
| CD56 | PerCP-Cy5.5 | HCD56 | BioLegend | 318321 |
| CD16 | FITC | 3G8 | BioLegend | 302006 |
| CD226 | APC | 11A8 | BioLegend | 338312 |
| CD52 | PE | HI186 | BioLegend | 316006 |
| NKG2D | PE/Dazzle™ 594 | S19004C | BioLegend | 375122 |
| CD62L | BV510 | DREG-56 | BioLegend | 304844 |
| CD69 | Pacific Blue^TM^ | FN50 | BioLegend | 310920 |
| KIR2DL1/S1/S3/S5 | PE-Cy7 | HP-MA4 | BioLegend | 339512 |
| Fixable Viability Stain 780 | APC-Cy7 | / | BD Pharmingen | 565388 |
| **NK cell subsets_functions** | | | | |
| CD3 | BV510 | OKT3 | BioLegend | 317332 |
| CD56 | PerCP-Cy5.5 | HCD56 | BioLegend | 318321 |
| CD16 | FITC | 3G8 | BioLegend | 302006 |
| IL-6 | PE | MQ2-13A5 | BioLegend | 501107 |
| Perforin | AF647 | dG9 | BioLegend | 308110 |
| IFN-γ | BV786 | 4S.B3 | BD Pharmingen | 563731 |
| GZMB | BV421 | GB11 | BioLegend | 515410 |
| Fixable Viability Stain 780 | APC-Cy7 | / | BD Pharmingen | 565388 |

**Supplementary Table 2. Correlations between the frequencies of NK cell subsets and clinical features of all the COVID-19 individuals.**

|  | NK | | CD56^bright^ CD16^-^ cells subset | | CD56^dim^ CD16^-^ cells subset | | NKeff cells subset (CD56^dim^ CD16^+^ NK) | |
| --- | --- | --- | --- | --- | --- | --- | --- | --- |
|  | *r* | *P value* | *r* | *P value* | *r* | *P value* | *r* | *P value* |
| Age | -0.058 | 0.58 | 0.176 | 0.09 | 0.150 | 0.15 | -0.196 | 0.06 |
| NEUT | 0.008 | 0.94 | 0.002 | 0.98 | -0.024 | 0.82 | 0.027 | 0.80 |
| LYM | -0.106 | 0.32 | -0.012 | 0.91 | -0.182 | 0.08 | 0.210 | 0.04 |
| PCT | 0.003 | 0.98 | -0.069 | 0.52 | 0.196 | 0.06 | -0.200 | 0.06 |
| CRP | -0.033 | 0.76 | -0.121 | 0.25 | 0.132 | 0.21 | -0.120 | 0.26 |
| IL6 | -0.080 | 0.45 | -0.149 | 0.16 | 0.367 | 0.0003 | -0.370 | 0.0003 |
| LDH | 0.019 | 0.86 | 0.119 | 0.26 | 0.111 | 0.30 | -0.146 | 0.17 |
| ALT | 0.033 | 0.76 | 0.015 | 0.89 | 0.115 | 0.28 | -0.136 | 0.20 |
| BUN | -0.159 | 0.13 | 0.008 | 0.94 | 0.100 | 0.35 | -0.103 | 0.33 |
| Length | 0.047 | 0.66 | -0.084 | 0.43 | 0.278 | 0.008 | -0.277 | 0.008 |

Correlation analyses between various NK cell subset frequencies and clinical features were performed by Pearson test.

Abbreviations: ALT, alanine aminotransferase; BUN, blood urea nitrogen; CRP, C-reactive protein; IL-6, interleukin-6; LDH, lactate dehydrogenase; Length: length of stay in hospital; LYM, lymphocytes; NEUT, neutrophils; PCT, procalcitonin.

**Supplementary Table 3. Detailed summary of glucocorticoid and antiviral administration (dose, timing, duration) for 37 patients.**

| Patient ID | Glucocorticoid | | | |  | Antiviral therapy | | | |
| --- | --- | --- | --- | --- | --- | --- | --- | --- | --- |
|  | Medication Name | Dose | Frequency | Duration (days) |  | Medication Name | Dose | Frequency | Duration (days) |
| P01 | Dexamethasone | 5mg | QD | 5 |  | Paxlovid | 300mg | Q12H | 8 |
|  |  |  |  |  |  | Ritonavir | 100mg |  |  |
| P02 | Methylprednisolon | 40mg | QD | 4 |  | Paxlovid | 300mg | Q12H | 8 |
|  |  |  |  |  |  | Ritonavir | 100mg |  |  |
| P03 | Methylprednisolon | 40mg | QD | 3 |  | Paxlovid | 300mg | Q12H | 8 |
|  |  |  |  |  |  | Ritonavir | 100mg |  |  |
| P04 |  |  |  |  |  | Azvudine | 5mg | QD | 3 |
| P05 |  |  |  |  |  | Paxlovid | 300mg | Q12H | 6 |
|  |  |  |  |  |  | Ritonavir | 100mg |  |  |
| P06 | Methylprednisolon | 40mg | QD | 3 |  |  |  |  |  |
| P07 |  |  |  |  |  | Paxlovid | 300mg | Q12H | 5 |
|  |  |  |  |  |  | Ritonavir | 100mg |  |  |
| P08 | Dexamethasone | 5mg | QD | 5 |  |  |  |  |  |
| P09 | Methylprednisolon | 40mg | QD | 3 |  |  |  |  |  |
| P10 | Methylprednisolon | 40mg | QD | 5 |  |  |  |  |  |
| P11 | Dexamethasone | 5mg | QD | 8 |  | Paxlovid | 300mg | Q12H | 8 |
|  |  |  |  |  |  | Ritonavir | 100mg |  |  |
| P12 |  |  |  |  |  | Paxlovid | 300mg | Q12H | 5 |
|  |  |  |  |  |  | Ritonavir | 100mg |  |  |
| P13 |  |  |  |  |  | Paxlovid | 300mg | Q12H | 5 |
|  |  |  |  |  |  | Ritonavir | 100mg |  |  |
| P14 | Methylprednisolon | 40mg | QD | 3 |  |  |  |  |  |
| P15 | Methylprednisolon | 40mg | QD | 2 |  |  |  |  |  |
| P16 | Methylprednisolon | 40mg | QD | 4 |  | Paxlovid | 300mg | Q12H | 8 |
|  |  |  |  |  |  | Ritonavir | 100mg |  |  |
| P17 | Methylprednisolon | 40mg | QD | 3 |  | Paxlovid | 300mg | Q12H | 9 |
|  |  |  |  |  |  | Ritonavir | 100mg |  |  |
| P18 | Methylprednisolon | 40mg | QD | 5 |  | Paxlovid | 300mg | Q12H | 5 |
|  |  |  |  |  |  | Ritonavir | 100mg |  |  |
| P19 | Methylprednisolon | 40mg | QD | 6 |  | Paxlovid | 300mg | Q12H | 8 |
|  |  |  |  |  |  | Ritonavir | 100mg |  |  |
| P20 | Methylprednisolon | 40mg | QD | 5 |  | Paxlovid | 300mg | Q12H | 7 |
|  |  |  |  |  |  | Ritonavir | 100mg |  |  |
| P21 | Methylprednisolon | 40mg | QD | 6 |  | Azvudine | 5mg | QD | 9 |
| P22 |  |  |  |  |  | Paxlovid | 300mg | Q12H | 5 |
|  |  |  |  |  |  | Ritonavir | 100mg |  |  |
| P23 |  |  |  |  |  | Paxlovid | 300mg | Q12H | 5 |
|  |  |  |  |  |  | Ritonavir | 100mg |  |  |
| P24 | Methylprednisolon | 40mg | QD | 7 |  | Paxlovid | 300mg | Q12H | 10 |
|  |  |  |  |  |  | Ritonavir | 100mg |  |  |
| P25 | Methylprednisolon | 40mg | QD | 6 |  |  |  |  |  |
| P26 | Methylprednisolon | 40mg | QD | 4 |  |  |  |  |  |
| P27 | Methylprednisolon | 40mg | QD | 7 |  | Paxlovid | 300mg | Q12H | 7 |
|  |  |  |  |  |  | Ritonavir | 100mg |  |  |
| P28 |  |  |  |  |  | Paxlovid | 300mg | Q12H | 9 |
|  |  |  |  |  |  | Ritonavir | 100mg |  |  |
| P29 | Methylprednisolon | 40mg | QD | 6 |  | Azvudine | 5mg | QD | 5 |
| P30 | Methylprednisolon | 40mg | QD | 5 |  | Paxlovid | 300mg | Q12H | 5 |
|  |  |  |  |  |  | Ritonavir | 100mg |  |  |
| P31 | Methylprednisolon | 40mg | QD | 4 |  |  |  |  |  |
| P32 | Methylprednisolon | 40mg | QD | 5 |  | Paxlovid | 300mg | Q12H | 5 |
|  |  |  |  |  |  | Ritonavir | 100mg |  |  |
| P33 | Methylprednisolon | 40mg | QD | 7 |  | Azvudine | 5mg | QD | 8 |
| P34 | Dexamethasone | 5mg | QD | 6 |  | Paxlovid | 300mg | Q12H | 7 |
|  |  |  |  |  |  | Ritonavir | 100mg |  |  |
| P35 | Prednisone | 20mg | QD | 5 |  | Azvudine | 5mg | QD | 5 |
| P36 | Methylprednisolon | 40mg | QD | 5 |  | Paxlovid | 300mg | Q12H | 6 |
|  |  |  |  |  |  | Ritonavir | 100mg |  |  |
| P37 | Methylprednisolon | 40mg | QD | 3 |  |  |  |  |  |

Abbreviations: QD, once daily; Q12H, every 12 hours.
